# Systematic target function annotation of human transcription factors

**DOI:** 10.1186/s12915-017-0469-0

**Published:** 2018-01-10

**Authors:** Yong Fuga Li, Russ B. Altman

**Affiliations:** 1 Stanford Genome Technology Center, Stanford, CA USA; 2 Department of Bioengineering, Stanford University, Stanford, CA USA; 3 Present address: Department of Bioinformatics, Illumina Inc., San Diego, CA USA; 4 Department of Genetics, Stanford University, Stanford, CA USA

**Keywords:** Transcription factor, Regulatory network, Gene function annotation, Functional pleiotropy, Regulator diversity, Target gene, Database, Function enrichment, Co-regulation

## Abstract

**Background:**

Transcription factors (TFs), the key players in transcriptional regulation, have attracted great experimental attention, yet the functions of most human TFs remain poorly understood. Recent capabilities in genome-wide protein binding profiling have stimulated systematic studies of the hierarchical organization of human gene regulatory network and DNA-binding specificity of TFs, shedding light on combinatorial gene regulation. We show here that these data also enable a systematic annotation of the biological functions and functional diversity of TFs.

**Result:**

We compiled a human gene regulatory network for 384 TFs covering the 146,096 TF–target gene (TF–TG) relationships, extracted from over 850 ChIP-seq experiments as well as the literature. By integrating this network of TF–TF and TF–TG relationships with 3715 functional concepts from six sources of gene function annotations, we obtained over 9000 confident functional annotations for 279 TFs. We observe extensive connectivity between TFs and Mendelian diseases, GWAS phenotypes, and pharmacogenetic pathways. Further, we show that TFs link apparently unrelated functions, even when the two functions do not share common genes. Finally, we analyze the pleiotropic functions of TFs and suggest that the increased number of upstream regulators contributes to the functional pleiotropy of TFs.

**Conclusion:**

Our computational approach is complementary to focused experimental studies on TF functions, and the resulting knowledge can guide experimental design for the discovery of unknown roles of TFs in human disease and drug response.

**Electronic supplementary material:**

The online version of this article (doi:10.1186/s12915-017-0469-0) contains supplementary material, which is available to authorized users.

## Background

Regulation of gene expression is essential for the realization of cell type-specific phenotypes [1] during normal development [2] and the adaptation of cellular organisms to their environment [3]. To a large degree, transcriptional regulation occurs through the interaction of protein factors with the genomic DNA [4]. Multiple proteins, including the chromatin remodelers, transcription factors (TFs), cofactors, and other transcription initiation factors [5], work in coordination to regulate the spatiotemporal details of gene expression. In the narrow sense, TFs are proteins that bind DNA in a sequence-specific manner and mediate the integrations of other proteins with specific target genes (TGs) for fine-granular expression control [6]. In this study, we adopt a broad definition of TF that includes the cofactors and other transcription initiation factors.

The pivotal role of TFs in development and cell identity determination is highlighted by the induced pluripotent stem cell (iPSC) technology [7, 8] and trans-induction techniques [9, 10], in which the introduction of just a few specific TFs is sufficient for converting fibroblast cells into pluripotent stem cells, or converting one differentiated cell type, e.g., pancreatic exocrine cells, directly into another differentiated cell type, e.g., β-cells. In addition, TFs are key players controlling diverse physiological functions, ranging from metabolism [11, 12], chemical and mechanical stress responses [13–16], song-learning [17, 18], to longevity and aging [19–21]. Many TFs are directly involved in diseases such as cancer, diabetes, and neural developmental disorders [9].

TFs have attracted intense research attention [22]; yet, the biological functions of most TFs are still poorly understood. The number of human TFs is estimated to be approximately 1500–2000 based on DNA-binding domain evidence [23–26]. In total, the sequence-specific DNA-binding activities of only 564 TFs are confirmed by experimental evidence and the existence of an additional 490 TFs is supported indirectly by phylogenetic evidence or author claims, based on the Gene Ontology (GO) database [27–30]. Limited knowledge is available on the biological functions of most TFs, with a small number of ‘famous’ TFs, such as TP53, attracting much attention [23]. However, recent developments of high-throughput technologies such as ChIP-seq and DNase-seq [31] provide an unprecedented amount of data on gene regulation, with binding profiles for over 100 TFs from ENCODE alone [32]. This has spurred systematic data-driven studies on transcriptional regulation, such as the discovery of cis-regulatory motifs [33, 34], the mapping of the hierarchical architecture of human gene regulatory networks, and the modeling of combinatorial regulation [32, 35–38]. At the same time, analytics tools have been developed for annotating ChIP-seq data [39–41], some allowing analysis of GO term enrichment for the binding sites [42–44].

**Fig. 1:**
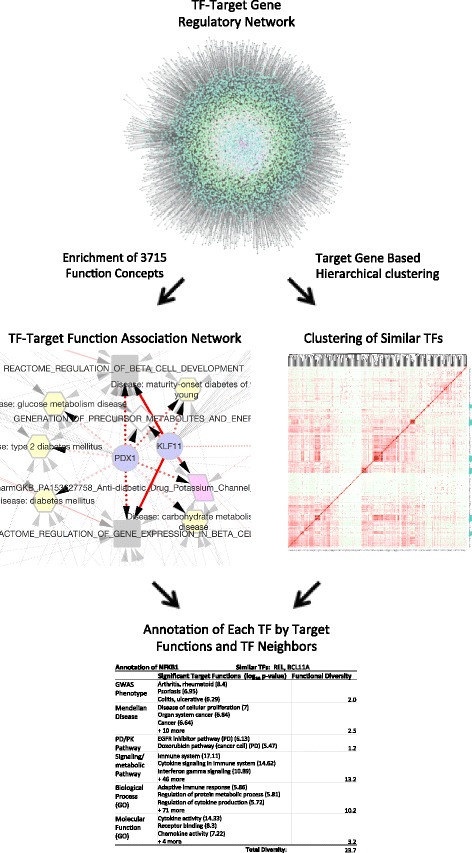
An outline of the workflow for regulatory network based annotation of transcription factor functions

In this study, we integrate the existing knowledge about functions and phenotypes of human genes with the transcriptional regulatory network to study the functions of human TFs. We define the ‘target functions’ of a TF as the statistical overrepresented functions among its TGs, and provide a systematic annotation of TF functions, ranging from metabolic pathways to disease phenotypes. In parallel, we define the functional similarity of two-TFs based on their TG overlaps, independent of the availability of gene function annotations, and annotate each TF by functionally similar TFs (Fig. 1). We study the pleiotropic functions of individual TFs and show that multifunctionality is associated with the number of upstream regulators of the TFs. With these analyses, we demonstrate a computational approach for achieving systematic understanding of TF functions.

## Results

### The compendium of human TF TGs

We compiled a TF–TG data compendium covering the direct transcriptional regulation targets of 384 unique TFs extracted from over 850 ChIP-seq experiments as well as the literature with low throughput experimental evidence. Low throughput experiments, ENCODE ChIP-seq, and other sources of ChIP-seq data are complementary in their TF coverage. It remains a challenge to accurately assign ChIP-seq signals to specific promoters and identify the TGs of a TF. We adapted a previously published method (TIP [45]), which statistically evaluates each gene as a potential TG based on both the locations and the intensities of the TF binding signals relative to the gene transcriptional start site(s). Overall, 149 (39%) TFs are covered only by high-throughput experiments, among which 52 (35%) are covered by the ENCODE consortium [32, 37] and 107 are covered by individual research labs (based on data published by October 2013). Meanwhile, 122 (32%) TFs are retrieved only from low-throughput experiments, and 113 (29%) TFs from both low- and high-throughput experiments (Additional file 1: Figure S1A).

A total of 16,967 unique TGs of TFs are available, including both TFs and non-TFs. We filtered the TGs identified in high-throughput experiments to achieve an estimated false discovery rate (FDR) of 0.01. Combining all sources, 146,096 TF–TG relationships were obtained. Each gene was regulated by 8.6 TFs in the compendium on average, while each TF in the compendium regulated 380.5 genes (Additional file 1: Figure S1B). Further, 63% of TGs were each regulated by five or more TFs, while 18% were each regulated by a single TF in the compendium. Most TFs also had regulators within the compendium, with the exception of 14 TFs that appeared to be master regulators among the TFs in the compendium, including BCOR, GLI2, HLF, HNF4G, MAZ, NELFE, NFATC1, NOTCH1, PHOX2A, RXRA, STAT4, SOX10, TEAD2, and THRA, although RXRA and SOX10 were self-regulated. Note that these TFs could be still be regulated by TFs without existing ChIP-seq data or regulated through distant cis-elements not effectively captured by current experimental/computational approaches.

### Defining the target functions of TFs

Transcription factors perform their functions by (1) interacting with proteins and cis-regulatory elements and (2) consequently regulating the expression of downstream TGs. There are hence two aspects of functions for a TF, the molecular functions of a TF that enables its regulation of the TGs, and the biological functions exerted by the genes that are under control of the TF. Formally, we define the target functions (e.g., target diseases, target signaling pathways) of a TF as the consensus functions of the TGs, and we identify the target functions of a TF by detecting the enrichment of functional terms in the TGs. The TGs, as a whole, precisely define the biological functions regulated by a TF, while the target functions summarize the functional impacts upon perturbation of a TF.

We first compiled 3715 functional concepts covering molecular to organism level functions (Additional file 1: Table S1), including Mendelian diseases from OMIM, disease and phenotype associations from dbGAP genome-wide association studies (GWAS), pharmacokinetic (PK) and pharmacodynamics (PD) pathways from PharmGKB, signaling and metabolic pathways from Reactome, and molecular functions and biological processes from GO. There were significant overlaps among the genes annotated in the six sources, yet each source has some unique genes (Additional file 1: Figure S2.)

**Fig. 2:**
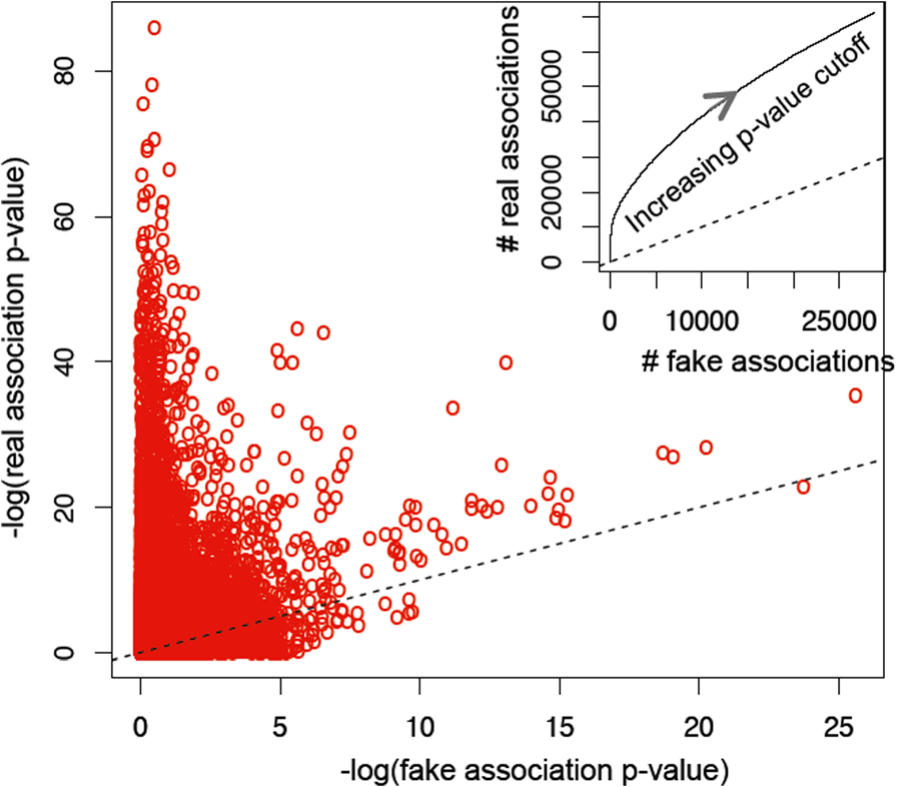
Presence of gene function signals in the TFTG data. The scatter plot shows the *P* values of function–TF associations obtained using real TFTG compendium (y-axis) and a fake TFTG compendium (x-axis). Each dot corresponds to a pair of *P* values for a TF–function pair. The inlet shows the number of significant TF–target function relationships at varying *P* value cutoffs for the real TFTG data (y-axis) against the number for the fake TFTG data (x-axis). *P* values were obtained by G-tests. Log base 10 was used

We then confirmed the presence of functional signals in the TFTG compendium, i.e., that TFs were not randomly targeting functionally unrelated genes, and that the TFTG compendium contained a significant number of true TGs. We compared the TF–function associations obtained using a real TFTG compendium against that obtained using a randomized compendium, where we constructed the fake TFs to have the same number of random TGs as the corresponding real TFs. We observed 237,566 TF–function pairs with *P* values for real TFs smaller than the corresponding *P* values for the fake TFs, compared to 155,801 pairs showing the opposite relationship (Fig. 2). To estimate the total number of true associations present for the real TFs, we assumed (1) that true associations for real TFs are all in the upper triangle, i.e., having *P* values from real compendium less than the corresponding *P* values from the randomized compendium and (2) that false associations for real TFs are equally distributed in the upper and lower triangle, i.e., having similar *P* values from the real and fake TFs. This led to an estimated 81,765 true target function annotations for the real TFs. The ratio between the true and false associations was larger at the smaller *P* value regions (Fig. 2 inset). At a *P* value cutoff of 0.001, there were 16,158 associations for real TFs and 999 for fake TFs, corresponding to an FDR of 6.18%; while at a *P* value cutoff of 0.0001, there were 9132 associations for real TFs but only 130 for fake TFs, corresponding to an FDR of 1.42%.

### Gene universe impacts the detection of target functions

The target functions of a TF are detected by identifying a statistically significant enrichment of functional terms among the TGs of the TF. A critical step to obtain proper statistics for enrichment analysis is the choice of gene universe, which is the ‘allowed’ set of genes that restrict the TGs of a TF as well as the member genes of a functional term to be used in determining statistical associations. In Additional file 1: Figure S3, we provide an example of TF SP1 and functional term ‘immune system’. The choice of gene universe affected not only the significance (*P* value) but also the direction of TF-target function association. In general, an overly large gene universe inflated the strength of the positive association, i.e., enrichment of functional terms, while an overly restrictive gene universe inflated the strength of the negative association, i.e., depletion of functional terms.

We suggest that the gene universe must be chosen based on the implicit limitations of each type of functional annotations stemming from how the annotation was obtained, thus generally providing a smaller and hence more conservative universe. For manual curation, such as OMIM and PharmGKB, the function annotations are limited by the available literature. We therefore constructed a conservative ‘Literature Rich’ gene universe that included protein-coding genes annotated by one or more sources from OMIM, PharmGKB, GO biological processes, GO molecular functions, Reactome, KEGG, and Biocarta. For machine annotations coming from high-throughput experiments followed by computational filtering, such as the GWAS phenotype annotations, we used the ‘coding genes’ as a conservative universe (see Methods for more details). We disregarded non-coding genes as they are generally poorly annotated. We used the Literature Rich gene universe to detect target Mendelian diseases, pharmacogenomic pathways, signaling/metabolic pathways, molecular functions, and biological processes, and used the coding gene universe to detect target phenotypes studied in GWAS.

### TF–target function network

**Fig. 3:**
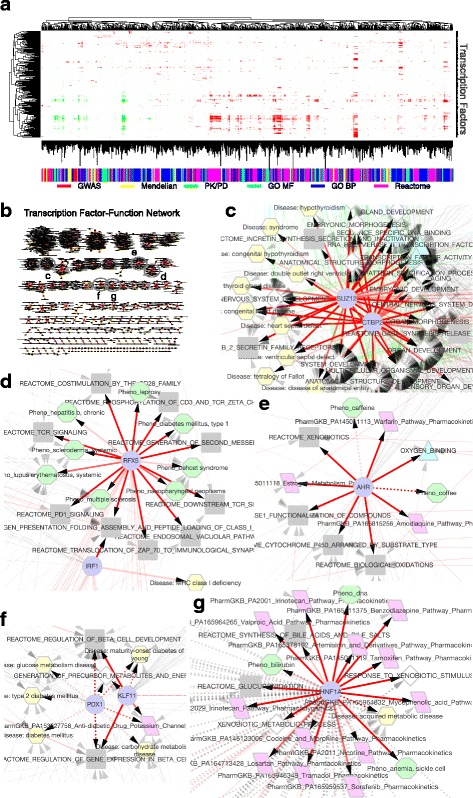
**a** Global view of the transcription factors (TFs) and their target functions; 311 TFs and 1420 annotations with one or more significant associations at FDR 0.1 levels were retained. Red indicates positive associations, green indicates negative associations, white indicates FDR > 0.1. Intensity of the colors corresponds to the significance levels: FDR 0.1, 0.05, and 0.01. The TF and target function clustering showed on the left and top was performed based on the TF-target function association phi coefficient matrix. We used the literature rich gene universe for the association analysis except for the TF-GWAS phenotype association, for which the coding gene universe was used. **b** The network visualization [148] of TF–target function and TF–known function relationships. Edges are colored red or green the same way as in (a). A solid edge links a TF with a significant target function that is not a known function. A dashed edge links a TF with a known function. A dashed edge with color links a TF with a known function that is also a significant target function, while a grey dashed edge links a TF with a known function that is not a significant target function. Node colors and shapes correspond to function types – purple circles, TFs; grey rectangles, Reactome pathways; blue triangles, GO molecular functions; white diamonds, GO biological processes; red rhomboids, PharmGKB PK and PD pathways; yellow hexagon, Mendelian diseases; green octagons, GWAS phenotypes. **c**–**g** Local regions of TF-function networks selected from **b**

At an FDR of 0.05, we identified 9747 significant TF–target function relationships using the conservative gene universes (Fig. 3a). The TF–target function associations formed a scale-free network [46], with power law distributions for both the number of target functions per TF and the number of TFs per target function (Fig. 3b and Additional file 1: Figure S4A). Overall, 279 (73%) TFs were annotated by at least one functional term (Additional file 1: Supplemental Material Section 1.1 [47, 48]). The lack of the annotations of the remaining TFs was likely due to the small sample size, i.e., number of TGs. The un-annotated TFs had 26.3 TGs on average, compared to 519.0 TGs on average for annotated TFs (Additional file 1: Figure S4B). An average TF was positively associated with 0.47 Mendelian diseases, 0.052 GWAS phenotypes or diseases, 0.26 pharmacogenomic pathways, 11.2 signaling and metabolic pathways, 7.9 biological processes, and 1.4 molecular functions (Additional file 1: Table S2). Extensive regulator sharing was observed among different types of gene functions (Additional file 1: Figure S5A), while we also observed biases of 62 TFs towards specific types of functions (Additional file 1: Figure S5B and Supplemental Material Section 1.2).

### Target functions predict known functions of TFs

We globally validated the TF–target function relationships by comparing them against the known functions of these TFs. Of course, our TF–target function relationships do not necessarily map to a TF–function relationship that is covered by existing gene annotation databases. For example, AHR targets molecular function *oxygen binding*, indicating that AHR regulates proteins that bind oxygen and likely catalyze oxidation reactions, but this does not mean oxygen binding is a molecular function of AHR protein itself. HNF1A targets many PK pathways (Fig. 3g), but HNF1A is naturally not an annotated member of these PK pathways, as the PK pathways in PharmGKB focus on the metabolic enzymes and transporters of drugs. Despite that, we found that the TF–target function associations could predict the known TF–target function relationships for all six types of functions. An overall area under the ROC curve (AUC) of 0.80 was achieved by using the *P* value from Fisher’s exact test as the predictive score. For specific types of functions, AUC of 0.81 was achieved for Mendelian diseases, 0.74 for GWAS phenotypes, 0.85 for pharmacogenetic pathways, 0.76 for GO biological processes, 0.76 for Reactome signaling and metabolic pathways, and 0.72 for GO molecular functions (Additional file 1: Figure S6). The true performance was likely higher, given the function–target function mapping issue.

Not only were target functions of TFs predictive of their known functions, but the numbers of target functions (i.e., multi-functionality) were also predictive of the numbers of known functions (Wald *t* statistic 6.07, *P* = 3.1 × 10^-9^; or Wald *t* statistic 5.07, *P* = 6.3 × 10^-7^ after controlling for the number of TGs per TF).

We manually validated the TF–target function relationships for Mendelian diseases, GWAS phenotypes, and pharmacogenetic pathways for which solid genetic evidence, such as direct mutation of the TF in patients, is available.

### Mendelian diseases targeted by TFs

We identified the target Mendelian diseases of a TF based on the enrichment of disease causing genes [49] in the TGs of the TF. In total, 181 TF–target Mendelian disease relationships were identified at a FDR of 0.05. Overall, the predicted relationships between TFs and target Mendelian disease strongly correlated with known genetic mutations of TFs in the target Mendelian diseases (two-sample Wilcoxon test *P* = 1.0 × 10^-159^). This suggests that the genetic architecture of human diseases reflects the structure of normal transcriptional regulatory network. The majority of the top 20 TF–Mendelian disease associations (from 13 TFs) were supported by direct genetic evidence such as mutations of the TF in the target Mendelian disease, GWAS associations between the TF and closely related diseases, or phenotypes closely related to the target disease as observed in mouse knockout models of the TF (Table 1). For example, we identified porphyria as a target disease of GATA1 (odds ratio 170, *P* = 9.8 × 10^-9^), while direct mutation of GATA1 (R216W) has been reported in a congenital erythropoietic porphyria patient [50], and the mutant was suggested to cause the disease by regulating UROS, a common causal gene of congenital erythropoietic porphyria. Details for more examples are available in Additional file 1: Supplementary Material Section 1.8.

**Table 1:** Top 20 TF-target disease associations. The “Literature Rich” gene universe is used for the association detection

TF	Target disease	log_2_(OR)^a^	*P* value^b^	Evidence^c^
ATF3	Lysosomal storage disease	4.5	3.9 × 10^-09^	–
BRCA1	Mitochondrial metabolism disease	3.1	3.7 × 10^-09^	–
CTBP2	Heart septal defect	6.9	4.4 × 10^-10^	Mouse^f^ [149]
	Congenital heart disease	6.5	1.6 × 10^-09^	Mouse^f^ [149]
ETS1	Organ system cancer	2.6	1.2 × 10^-08^	Mutation^d^ [150]
GATA1	Acute porphyria	7.4	9.8 × 10^-09^	Mutation^d^ [50]
HNF4A	Mitochondrial metabolism disease	3.0	5.3 × 10^-09^	Mutation in MODY1^d^ [140]
NFE2	Lysosomal storage disease	5.7	4.8 × 10^-12^	–
	Lipid storage disease	6.0	5.8 × 10^-09^	–
RFX2	Bardet-Biedl syndrome	5.5	1.1 × 10^-12^	Mouse^f^ [144, 145]
SOX10	Waardenburg’s syndrome	11.4	2.0 × 10^-09^	Mutation^d^ [141]
SUZ12	Heart septal defect	6.2	7.4 × 10^-09^	Mouse^f^ [151]
TP53	Organ system cancer	3.5	1.5 × 10^-19^	Mutations in multiple cancer^d^
	Cancer	3.5	3.6 × 10^-19^	Types [152]
	Disease of cellular proliferation	3.4	1.7 × 10^-18^	
	Reproductive organ cancer	4.8	1.2 × 10^-08^	
USF1	Disease of metabolism	2.2	1.3 × 10^-10^	Association with FCHL^e^ [153, 154]
	Inherited metabolic disorder	2.2	7.5 × 10^-09^	
USF2	Lysosomal storage disease	4.1	1.3 × 10^-09^	–
	Disease of metabolism	2.3	2.8 × 10^-09^	–

^a^log2(OR), log2 transformed odds ratio

^b^*P* value from single-tailed Fisher’s exact test for odds ratio > 1

^c^Evidence lists published genetic evidence directly support the association of the TF with the disease

^d^Mutation mutations of the TF are observed in the disease or closely related diseases

^e^Association, the TF gene locus is genetically associated with the disease or related diseases

^f^Mouse mouse model shows phenotypes directly related to the disease.

Non-genetic evidence in the literature is not considered.

*MODY1* maturity-onset diabetes of the young, *FCHL* familial combined hyperlipidemia

**Table 2:** Discordance transcription factors’ target function similarity and target gene similarity

TF pair classification			Counts	Significant sharing of known functions^c^	*P* value
Description	Target-function sharing	Target-gene sharing			
Unexpected target function similarity	Significant^a^	Low	329	117 (35.6%)	0.0010 OR = 1.45
Other pairs with low target gene sharing	Not	Low	42,373	11,704 (27.6%)	
Expected target function similarity	Significant^a^	High^b^	4583	1772 (38.7%)	7.7 × 10^-13^ OR = 1.27
Other pairs with high target gene sharing	Not	High^b^	26,251	8727 (33.2%)	

^a^Significant target-function sharing: target function overlap significantly higher than expected by change (FDR ≤ 0.05)

^b^High sharing of target gene: odds ratio of target gene sharing between a pair of TF is ≥ 1

^c^Significant sharing of known functions: known function overlap significantly higher than expected by change (FDR ≤ 0.01, see Additional file 1: Table S9 for results at threshold 0.05)

*OR* odds ratio

### Complex phenotypes targeted by TFs

We identified 20 significant complex phenotypes for seven TFs (Additional file 1: Table S3 [51–62]). Transcription factors NFKB1 and RFX5 (Fig. 3d) are each associated with three and six autoimmune disorders, while both TFs are known to be involved in autoimmunity [53, 63, 64]. Especially, NFKB1 has been recently identified as a causal gene of autosomal dominant variable immunodeficiency-12 [65], which shows features of autoimmunity. NFKB1 is also genetically associated with autoimmune disease Ulcerative colitis [61]. Details of additional TF–target phenotype relationships are available in Additional file 1: Supplementary Material Section 1.9.

### Pharmacogenetic pathways targeted by TFs

We identified 99 TF–target pharmacogenomic pathway relationships, covering 47 unique TFs and 45 unique pharmacogenetic pathways in PharmGKB. There was no preference towards PK or PD pathways, with 20 of 40 PK pathways and 26 of 50 PD pathways identified. However, different TFs were responsible for the target PK and PD pathways. Further, 18 of the 26 target PK pathways were the targets of just four TFs (see Additional file 1: Table S4), i.e., HNF1A, AHR, NR1I3, and FOXA2. Among them, nuclear receptor genes HNF1A, AHR, and NR1I3 are well known to regulate xenobiotic-metabolizing enzymes [60, 66–68]. Unique target PK pathways were found for each of the four TFs, suggesting their complementary roles in regulating drug metabolism. In addition to these four TFs, SP1 and TP53 were each associated with three PK pathways for cancer drugs. SP1 and TP53 were also associated with other cancer PD pathways, and their associations with cancer are strongly supported by the literature [69, 70].

We manually examined the full list of identified target PD pathways and confirmed the majority of the associations (Additional file 1: Table S4). A PD pathway describes the disease pathway that is perturbed by a drug. A target PD pathway is considered confirmed if the TF is a member of the PD pathway or closely related pathways, or if the TF is known to be genetically linked to the disease or closely related phenotypes. For example, ELK1 is identified as a regulator of the EGFR Inhibitor Pathway, while the TF itself is a member of the PD pathway. HNF1A is identified as a regulator of the PD pathways for cancer, high cholesterol, and diabetes, while mutations of HNF1A are known to cause hereditary cancers and diabetes, and variants of HNF1A are strongly associated with cholesterol level in GWAS [71]. E2F1 and E2F4 have been identified for multiple antimetabolite PD pathways. Antimetabolites are a class of drugs used for inducing medical abortions and treating cancers and autoimmune diseases through halting of cell cycles, while E2F1 and E2F4 are well-known regulators of cell cycles [72, 73].

### TG sharing among TFs

While the TGs of a TF define its biological functions, the TG sharing between two TFs also reflects the functional relatedness between TFs. We studied the relationship between the TG overlaps and target function sharing between pairs of TFs.

As expected, the TG sharing, measured by Pearson’s phi coefficient *ϕ*_*TG*_, was highly associated with the target function sharing *ϕ*_*Target Fun*_ (Wald *t* statistic 126.95, or 109.75 when controlling for the number of TGs, both *P* < 2.2 × 10^-16^). Among 73,536 possible TF pairs (Additional file 1: Figure S7), 12,434 (16.9%) showed significant TG sharing at a FDR of 0.01 based on Fisher’s exact test. We refer to these similar TFs as TF neighbors. Relatedly, there were 11,205 pairs of TFs with one or more shared target functions, including 5866 pairs that also showed significant TG sharing (odds ratio 9.3).

Despite the overall consistency between target function overlap and TG sharing, many exceptions occur. Significant TG sharing was observed for 6568 pairs of TFs that did not share any target functions, including 428 pairs that surprisingly showed negative correlations between their target function association profiles.^1^ This could be caused by unknown or poorly understood functions common to these TF neighbors, and suggests that the TG-based TF neighbors may provide functional information missed by the target functions; therefore, the TF neighbors may serve as an additional layer in the TF function annotations. On the other hand, significant target function sharing (at a FDR < 0.05) was observed for 329 pairs of TFs that had lower-than-expected TG overlaps. To validate these unexpected target function similarities, we examined the known (literature reported) functions of these TF pairs. Indeed, 35.6% [74] out of the 329 pairs are supported by the literature, compared to 27.6% of the other TF pairs (*P* = 0.001 by Fisher’s exact test, see Table 2). The top five TF pairs by target function sharing were MXI1 and RFX1, TRIM28 and VDR, LMO2 and ZNF263, ARNTL and BHLHE40, and ETV5 and MXI1. Among these, two pairs, TRIM28 and VDR, and ARNTL and BHLHE40, did not share any TGs. However, TRIM28 and VDR shared 12 target functions, e.g., *Reactome Telomere Maintenance*, out of 15 and 14 target functions for the two TFs, respectively; while ARNTL and BHLHE40 shared two target functions, *Reactome Bmal1 Clock Npas2 Activates Circadian Expression* and *Reactome Circadian Clock*, out of four and two target functions for the two TFs, respectively. Mouse gene knock-out confirmed an abnormal circadian rhythm as a phenotype for both ARNTL [75, 76] and BHLHE40 [77], and the two proteins may be interaction partners [78]. A complete list of the TF pairs is available in Additional file 4: Table S8.

### TFs link apparently unrelated functions: coffee and warfarin

Parallel to the TF–TG sharing and TF–target function sharing, we observed extensive member gene overlaps and regulator sharing between pairs of functional concepts (Additional file 1: Supplemental Material Section 1.3). The majority of TFs (64%) had two or more target functions. We observed that apparently unrelated gene functions were frequently linked by TFs. For example, AHR was found to be associated with coffee consumption and the PK pathways for drugs amodiaquine, warfarin, erlotinib, and phenytoin, as well as the estrogen metabolism pathways (Fig. 3e). Based on these observations, we hypothesized that coffee consumption would interfere with the metabolism of these drugs and estrogen, either through modifying the activities of AHR target enzymes or by impacting the expression of the enzyme genes through feedback regulation of AHR activity. The interactions of coffee drinking with both warfarin [79] and phenytoin [80] have been reported. On the other hand, coffee consumption is actually associated with decreased venous thromboembolism [81], which warfarin can effectively treat. The coffee–estrogen link is even more intriguing. High coffee intake has been found in multiple studies to be significantly associated with decreased risk of estrogen receptor-negative breast cancer [82, 83] and breast cancer risk in *BRCA* mutant carriers [84]. In addition, high coffee intake impacts the risk of Parkinson’s disease in female in an estrogen-dependent manner [85, 86], possibly through modifying blood estrogen levels [87].

Indeed, we believe two apparently unrelated functions or phenotypes can be inherently related, and the relationship can be discovered through the TG based on TF function annotation as performed herein.

**Fig. 4:**
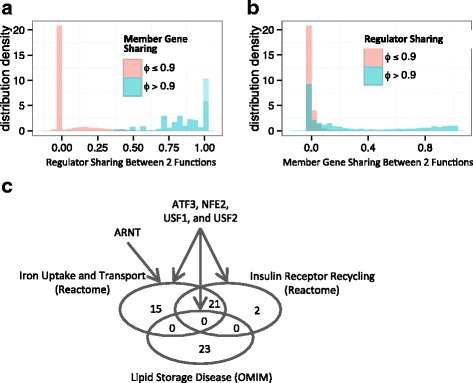
Transcription factor sharing among apparently unrelated functional concepts. **a** Two functional concepts with high member gene overlaps always have similar regulators, but (**b**) two functional concepts with nearly identical regulators do not always have high member gene sharing. **c** A Venn diagram for three functional concepts for which shared transcription factors are identified for functions without gene overlaps. The arrows connect the significant regulators for the functions. Note that *Iron Uptake and Transport* and *Insulin Receptor Recycling* do share member genes significantly, but neither of them shares member genes with *Lipid Storage Disease*

Obviously, when two functional concepts are statistically associated, i.e., when they share a significant number of member genes, they will likely be linked to the same regulators (Fig. 4a and Additional file 1: Figure S9A); however, the inverse is not true. Two functions can be linked by TFs even when they do not share a significant portion of member genes (Fig. 4b and Additional file 1: Figure S9B). In fact, of the 954 function pairs that shared identical sets of regulators, 356 (37%) pairs had less gene overlap than expected by chance (Additional file 2: Table S6), i.e., with odds ratio < 1. Most of such function pairs did not share any member genes. For example, hereditary *lipid storage diseases* did not share any genes with Reactome pathways *iron uptake and transport* and *insulin receptor recycling*, but the three functions were found to share regulators ATF3, NFE2, USF1, and USF2, while *iron uptake and transport* was also a target function of ARNT (Fig. 4c). Other examples include *ventricular septal defect* and developmental *pattern specification process*, which are both targeted by SUZ12 and CTBP2, *PECAM1 Interactions* and disease *agammaglobulinemia* targeted by EBF1, *intestinal disease* and *Human immunodeficiency virus infectious disease* both targeted by NFKB1, *prostate cancer* and *intestinal cancer* both targeted by TP53, and *Metalloendopeptidase Activity* and *cognitive disorder* both targeted by ETV4, among many others.

### Measuring the functional pleiotropy of TFs

**Fig. 5:**
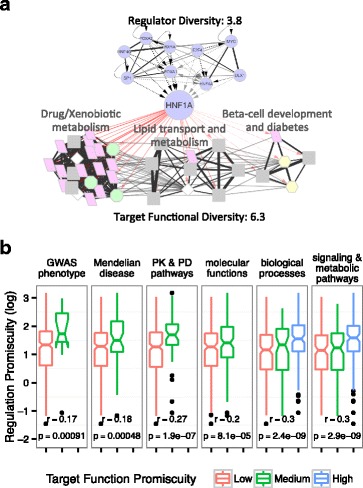
The relationship between functional diversity and regulator diversity of transcription factors (TFs). **a** The target functions of TF HNF1A form three major clusters based on similarities (member gene sharing) among the functions, while the upstream regulators of HNF1A form clusters based on the functional similarities (target gene overlaps) among these regulators. The regulator and functional diversities of a gene measures the effective number of regulators and effective number of functions for a gene. The coloring schema is the same as in Fig. 3 and the clustering of TFs and functions are based on the TF’s target gene overlaps and the function’s member gene overlaps. **b** Significant associations exist between the regulator diversity and target function diversity of TFs for six types of function annotations separately

A TF is functional pleiotropic if it targets multiple unrelated functions. The above analyses suggest extensive functional pleiotropy of TFs. In addition, while examining the functional pleiotropy of TFs, we observed that it was correlated with the regulator diversity of the TFs. Here, we quantified the functional pleiotropy of TFs in order to further study, in the next section, its causes from the perspective of transcriptional regulation. The number of target functions *n*_*Target Fun*_ can be a measure of TF functional pleiotropy, with the caveat that it double counts closely related or redundant functional concepts. We hence defined function diversity *π*_*Target Fun*_ as the ‘effective’, i.e., non-redundant, number of target functions by weighting each function by its uniqueness, which is the inverse of the accumulative similarity between the function and other functional concepts. Similarly, we define regulator diversity *π*_*Reg*_ of a gene as the effective, i.e., non-redundant, number of regulators. The regulator diversity corrects for related or cooperative TFs that are counted independently in the number of regulators *n*_*Reg*_ targeting a gene (Methods and Additional file 1: Supplemental Material Section 1.4). To motivate further analysis, we present examples of TFs with different levels of functional pleiotropy and regulator diversity in Table 3 as well as in Figs. 5a, Table 4, and Additional file 1: Figure S11A.

**Table 3:** Functional pleiotropy and regulator diversity of selected transcription factors (TFs), including two most functional pleiotropic TFs, BRCA1 and ZNF143, two TFs with the highest upstream regulatory diversity, MYC and TP53, and three TFs with lower functional pleiotropy, HNF1A, NFKB1, and SUZ12

TF	Function pleiotropy				Regulator diversity	
	Target function	Effective target function	Known function	Effective known function	Upstream regulator	Effective upstream regulator
BRCA1	272	45.5	101	14.5	33	15.9
ZNF143	242	35.4	22	2.8	53	22.1
MYC	159	24.2	68	12.4	50	25.1
TP53	175	26.8	166	25.4	49	23.0
HNF1A	30	6.3	58	11.2	9	3.8
NFKB1	143	23.7	34	6.2	26	11.7
SUZ12	48	8.3	4	1.0	11	4.9

**Table 4:** Complete target gene-based annotations for two example transcription factors (TFs) (A) NFKB1 and (B) SUZ12. Three types of information are provided (1) the top TF neighbors obtained by TF distance (target-gene overlap measured by Pearson’s phi coefficient) < 0.8, (2) the target functions in six categories, and (3) the functional diversities in six categories and total diversity. See Additional file 1: Figure S9 for a visualization of the regulator and target function networks surrounding NFKB1

A		
**Annotation of NFKB1**	**Similar TFs**: REL, BCL11A	
	**Significant target functions** (**log**_**10**_ *** P *** **value**)	**Functional diversity**
GWAS phenotype	Arthritis, rheumatoid (8.4) Psoriasis (6.95) Colitis, ulcerative (6.29)	2.0
Mendelian disease	Disease of cellular proliferation (7) Organ system cancer (6.84) Cancer (6.64) + 10 more	2.5
PD/PK pathway	EGFR inhibitor pathway (PD) (6.13) Doxorubicin pathway (cancer cell) (PD) (5.47)	1.2
Signaling/metabolic pathway	Immune system (17.11) Cytokine signaling in immune system (14.62) Interferon gamma signaling (10.89) + 46 more	13.2
Biological process (GO)	Adaptive immune response (5.86) Regulation of protein metabolic process (5.81) Regulation of cytokine production (5.72) + 71 more	10.2
Molecular function (GO)	Cytokine activity (14.33) Receptor binding (8.3) Chemokine activity (7.22) + 4 more	3.2
	**Total Diversity:**	23.7
B		
**Annotation of SUZ12**	**Similar TFs**: CTBP2	
	**Significant Target Functions** (**log**_**10**_ ***P *** **value**)	**Functional diversity**
GWAS phenotype	–	0
Mendelian disease	Heart septal defect (8.13) Congenital heart disease (7.57) Disease (7.14) + 6 more	1.8
PD/PK pathway	–	0
Signaling/metabolic pathway	Regulation of beta cell development (12.19) Regulation of gene expression in beta cells (6.26) Class b 2 secretin family receptors (4.08)	1.2
Biological process (GO)	Anatomical structure development (40.03) Multicellular organismal development (33.22) System development (32.56) + 29 more	5.8
Molecular function (GO)	Transcription factor activity (36.68) DNA binding (33.55) RNA polymerase II transcription factor activity (11.83) + 1 more	2.2
	**Total Diversity:**	8.3

### Upstream regulation enables functional pleiotropy of TFs

Over the set of 384 TFs in the TFTG compendium, we observed a global positive association between the target function diversity of TFs with the regulator diversity (Wald test *P* = 3.3 × 10^-10^ between diversity measures *π*_*Target Fun*_ and *π*_*Reg*_, or *P* = 1.6 × 10^-9^ between raw counts *n*_*Target Fun*_ and *n*_*Reg*_), i.e., TFs with more effectively unrelated upstream regulators also tended to have more effectively unrelated target functions, suggesting diverse modes of upstream regulation as a mechanism for TFs to realize functional pleiotropy. To eliminate technical biases due to ChIP-seq experiment quality or uneven research attention for different TFs, we controlled for *n*_*TG*_, the number of TGs per TF, as a confounding factor through a linear model. However, regulator diversity remained a significant predictor of the TF’s function diversity (*P* = 5.3 × 10^-6^, Wald test). Further, we examined the known functions of TFs, which, unlike the target functions, were independent of the TFTG data compendium. A significant association remained between the known function diversity and the regulator diversity of TFs (*P* = 6.3 × 10^-5^ between diversity measures *π*_*Known Fun*_ and *π*_*Reg*_, or *P* = 0.00022 between raw counts *n*_*Known Fun*_ and *n*_*Reg*_). This was true regardless of the number of TGs for the TFs. In fact, a slightly stronger correlation was observed when TFs with less than 100 TGs were removed (Additional file 1: Figure S12). Finally, to completely eliminate the impact of human research biases toward popular TFs, which could result in a higher number of literature-reported TGs as well as literature-reported upstream regulators for the popular TFs, we repeated all of the above experiments after removing all low-throughput (literature derived) data in the TFTG compendium. We observed that regulator diversity and function diversity remained significantly associated (Additional file 1: Supplemental Material Section 1.5). As a control, we evaluated the association between the TF’s functional pleiotropy and its hierarchical location within the gene regulatory network, measured by PageRank [88]. Neither the PageRank-function diversity nor the PageRank-target function diversity associations were significant after controlling for the number of TGs of TFs (Additional file 1: Supplemental Material Section 1.6 [89, 90]).

In addition, we observed that the positive association was universal for all six types of function annotations. The trends were stronger for biological processes and molecular pathways, and weaker for GWAS and disease phenotypes (Fig. 5b). The association between function and regulator diversities extended to non-TF genes as well, with *P* = 7.9 × 10^-5^ between diversity measures *π*_*Fun*_ and *π*_*Reg*_, and *P* = 3.0 × 10^-18^ between raw counts *n*_*Fun*_ and *n*_*Reg*_ for 11,345 genes that have both regulator and function annotations (Additional file 1: Supplemental Material Section 1.7).

If regulator diversity is indeed a cause of TF function diversity, it is likely through driving the expression of the TF in diverse conditions. To evaluate this mechanism, we examined the expression of TFs in a collection of 327 human tissue types and cell lines [91]. As expected, expression diversity of TFs was significantly associated with the regulator diversity (Spearman rank correlation 0.22, *P* = 2.7 × 10^-6^, or Spearman rank correlation 0.26, *P* = 3.6 × 10^-7^ for the raw counts). On the other hand, there was a significant association between expression diversity of TFs and the target-function diversity (Spearman’s rank correlation 0.10, *P* = 0.048) and the function diversity (Spearman’s rank correlation 0.26, *P* = 2.2× 10^-7^). Similarly, we observed strong associations between expression diversity of general genes and the function and regulator diversities of genes (Additional file 1: Supplemental Material Section 1.7). These findings support transcriptional regulation diversity as a mechanism for functional pleiotropy of TFs and other genes.

## Discussion

A major challenge in data-driven TF function annotation is to minimize the impacts from false bindings and to reliably extract gene function signals. We combined multiple statistical strategies to achieve this. First, TGs from ChIP-seq experiments were extracted with a stringent FDR, which was calculated using a statistical framework modified from TIP [45] by combining binding locations and intensity information to enrich for true TF-DNA binding events over false signals. Second, we defined the target functions of TFs as the consensus functions among the putative TGs. The statistical enrichment analysis hence further filtered noises from the remaining false TGs. Third, we chose conservative gene universes specific to the types of functions, so as to minimize spurious associations. Finally, we applied the Benjamini–Hochberg multi-test correction procedure and required a FDR of 5% for all associations reported. With these, approximately 10,000 significant TF-target function associations were obtained. Meanwhile, the total number of true TF-target function associations was estimated to be over 80,000, indicating the presence of rich functional signals in the TFTG data (Fig. 3). We believe there is room for further improvement to retrieve a higher number of TF-target function annotations at a controlled FDR.

We globally validated the TF-target function associations by comparing them with known TF-function relationships, and showed that the target functions cover both known and novel TF-function relationships. Despite the fact that TF-target function and TF-function relationships did not always have direct correspondence, we observed a good prediction performance with an AUC 0.80 with six types of gene functions combined. In addition, we manually validated the top target diseases, phenotypes, and pharmacogenetic pathways based on the literature, and found the majority to be supported by direct genetic evidence, such as direct mutations or GWAS implicated associations of a TF in patients with the target disease, or phenotypes of mouse knock-out models of the TF (Table 1, Additional file 1: Tables S3 and S4 [49, 71, 92–115]), even when they were not annotated as a known function of the TFs. Given that our knowledge was incomplete for even the most well studied TFs, we believe the non-validated TF-target functions represent opportunities for future experimental studies of the TFs.

The foundation of this study was the hypothesis that genes regulated by a same TF are functionally related. We believe this extends to the functional concept level, i.e., that multiple concepts targeted by the same TF(s) are also functionally related at some higher level. Based on co-regulation, we predicted the interaction between coffee consumption and the metabolism of multiple drugs, including warfarin, as well as the interaction between coffee consumption and estrogen metabolism, both of which are validated by multiple published experimental studies [79–86]. Further, we showed that TFs link hundreds of functional concept pairs that do not share any member genes. This highlights the potential usage of the TF-target function network to study the high-level organization principles among biological functions that is unattainable by solely studying the member genes of functions, e.g., through a member gene-based function–function association network.

Based on the TF-target function network, we examined the functional pleiotropy of TFs. We discovered that a TF with more target functions (or known functions) were themselves regulated by significantly more TFs, and both function and regulator diversities were associated with the expression diversity of the TF in cell lines and tissues. These findings suggest that regulator diversity may be a cause of function diversity of TFs, and it works by driving the expression diversity of genes.

TF–TG interactions mediated by distant cis-regulatory regions, e.g., enhancers, are challenging to identify due to the large variations in the relative locations of enhancers. Such signals are not captured in this study. In an attempt to capture distant regulations, we relaxed the window size from 6000 to 20,000 bps in the statistical inference of TF-target genes (Additional file 1: Supplemental Methods section 2.1). We observed that the majority of the TF–TG relationship remained the same. Given that the statistical signal is expected to be weaker for bindings at larger distance to TSS, the existing experimental and computational frameworks are in general inefficient in capturing enhancer regulations. In addition, an overly wild window would reduce statistical power in detecting the true signals. This study therefore focused on the smaller 6000-bp window.

Gene regulation is well known to be cell type-specific, and co-expression of TFs is required for the co-regulation of TFs on the shared TGs [24]. However, current high-throughput studies for in vivo TF-DNA binding, including the ENCODE project [32, 116], are generally limited to a small number of tissue/cell types. Comprehensive ChIP-seq analysis on a large number of cell types remains unrealistic due to cost and resource requirements. We therefore compiled the TF–TG relationships in a cell type- and development stage-agnostic manner. Contingent on data availability, this work can be easily extended to perform cell type-specific TF function annotation. Despite this limitation, the resulting TFTG data partially captured the cell type specificity of TFs, as we observed that TFs sharing similar tissue expression patterns also shared a greater amount of TGs (Wald *t* test, *P* = 1.9 × 10^-78^).

## Conclusion

In an effort to manually annotate TF functions [22], over 100 experts joined efforts to curate and integrate published knowledge and provide mini-reviews on TFs. We believe automated yet accurate function annotation and manual curation are complementary and will together greatly facilitate our understanding of the biological functions of human TFs.

Despite large consortium efforts such as ENCODE [32, 117], existing data for TF–TG relationships remains scarce. Our TFTG compendium covers 384 unique TFs. This is the largest collection, to our knowledge, compared to 237 TFs in a recently published study [74], yet it only covers a small fraction (20–25%) of the putative 1500–2000 TFs in human [23, 26]. Relatedly, we notice that the TFTG compendium is biased toward the well-known TFs, likely due to preferential attachment of research efforts to popular TFs. For the same reason, some TFs enjoy higher TG coverage than the others. These biases currently limit the power of TG-based TF function annotation. However, with the maturity of ChIP-seq and related high-throughput assays for in vivo protein-DNA binding and the availability of the technologies to more labs, we expect a steady accumulation of TFTG data with improved accuracy and completeness, yet with reduced biases. Such data will ultimately enable the annotation of all TFs in the human genome, and serve as the foundation for hypothesis generation and further experimental studies of the roles of TFs in normal biological processes and diseases.

## Methods

### Transcription factor TG data compendium

We compiled TFTG relationships from multiple sources. ChIP-seq experiments from both large- [28, 32, 37] and small-scale studies were included. Meta-data of 413 ChIP-seq experiments for 235 unique TFs were curated manually by October 2013 from GEO [118], in addition to approximately 450 ChIP-seq experiments for 115 unique TFs from the ENCyclopedia of DNA elements (ENCODE) [32, 37]. The binding signals from TGs were differentiated from those from non-TGs using a modified version of the TIP algorithm [45], which combines the binding location and intensity information for statistical determination of TF TGs (Additional file 1: Supplemental Methods section 2.1 [119–121]). Manually curated low-throughput TG annotations were compiled from multiple databases, including BIND, HTRI, PAZAR, and TRED [122–125]. Only TFTG relationships with direct literature evidence from low-throughput experiments [126, 127], e.g., as electrophoretic mobility shift assays, were included. We did not differentiate sequence-specific DNA-binding TFs from other DNA binding transcriptional regulators. Some cofactors that do not directly bind DNA were also included when ChIP-seq data were available. Despite this, we refer to all these transcriptional regulators as TFs in this study.

### Gene function annotation data

Six types of gene annotations were used in this analysis to annotate TFs. GO [25] for biological processes and molecular functions, together with the Reactome pathways [128] were retrieved from the MSigDB v4.0 [129]. The pharmacogenomics pathways for PD and PK were retrieved on January 20, 2013, from the Pharmacogenomics Knowledgebase (PharmGKB) [130]. Gene disease association data from GWAS were obtained on May 4, 2014, from dbGAP [131] and NHGRI [132] catalogs with *P* value cutoffs at 1 × 10^-3^ (loose set) or 1 × 10^-5^ (stringent set), and the closest gene (or two genes if the SNP was intergenic) to each SNP was retained. When not specified, the loose set was used. Of note, a large *P* value cutoff was used to capture the majority of the true disease-related genes rather than to select for confident ones, as our goal here was to associate complex phenotypes and diseases rather than individual genes with TFs. The gene–Mendelian disease annotations were obtained on July 5, 2014, from the Online Mendelian Inheritance in Man (OMIM) [49], and the disease genes were further grouped in a hierarchical manner to disease classes based on the disease ontology [133]. For all data, only genes uniquely mapped to the Entrez Gene database [134] were retained.

### Defining coding genes and Literature Rich genes

Coding genes were defined as all Entrez genes that have associated protein products in Ensembl Protein or UniProt databases. Literature Rich genes were defined as coding genes annotated in any of the following seven data sources: GO Biological Processes, GO Molecular Functions, Reactome, PharmGKB, Kyoto Encyclopedia of Genes and Genomes pathways [135], Biocart [136], and OMIM [49]. There were 19,847 coding and 10,931 Literature Rich genes in total. Interestingly, 333 of the Literature Rich genes were not Coding genes, but pseudogenes, discontinued gene records, or gene loci without defined genes. These were removed, leaving 10,561 Literature Rich genes in total.

### Measuring the associations between binary variables

Fisher’s exact test [137] was used for testing the associations between TFs and biological functions by detecting significant enrichment of genes that were TGs of a TF and were also annotated with a given function. G-test was used as a fast approximation to Fisher’s exact test in preliminary analyses and to demonstrate the presence of functional signals in the TF TG data (Fig. 3). To perform multi-test correction, we calculated the Benjamini–Hochberg FDR [138] on the *P* values for each type of annotation separately.

Since Fisher’s exact test does not have a test statistic that can be used to measure the similarities between two binary variables, we used Pearson’s phi coefficient (*ϕ*, PPC) to measure association strength,

$$ \phi =\frac{n_{11}{n}_{00}-{n}_{10}{n}_{01}}{\sqrt{\left({n}_{10}+{n}_{11}\right)\left({n}_{00}+{n}_{10}\right)\left({n}_{01}+{n}_{11}\right)\left({n}_{00}+{n}_{01}\right)}}, $$

where *n*_*ij*_ are the observed number of *ij* value pairs for the two random variables. The strengths of TF–function association, TF–TF TG sharing, TF–TF target function sharing, TF-TF known function sharing, and function–function member gene sharing are denoted as *ϕ*_*TF*_*Fun*_, *ϕ*_*TG*_, *ϕ*_*TargetFun*_, *ϕ*_*Fun*_, and *ϕ*_*Fun*_*Fun*_ respectively. PPC is sample size independent, and serves as a good measure of the magnitude of associations. The sign of PPC indicates the directionality of an association.

### Functional and regulator diversities of TFs

We measured the effective number of TFs (i.e., the regulatory diversity) of a function or gene and the effective number of target functions (i.e., the function diversity) of a TF by down weighting the TFs (or functions) that were correlated with other TFs (or functions). Given Pearson’s phi coefficient ${\phi}_{t{t}^{\prime }}$ between TFs *t* and *t*^'^, the uniqueness of TF *t* is defined as ${u}_t=1/{\sum}_{t^{\prime}\in TFs}{\phi}_{t{t}^{\prime}}^2$. Note that *u*_*t*_ is always within 0 to 1, since the association between a TF with itself is always 1, i.e., *ϕ*_*tt*_^2^ = 1. The regulator diversity *π*_*Reg.g*_ of a function or gene (including TF) *g* is then defined as the weighted counts of the TFs targeting the function or gene, *π*_*Reg.g*_ = ∑_*t* ∈ *TFs regulating g*_*u*_*t*_. The regulator diversity measures the effective (non-redundant) number of regulators for a gene (or a TF). Similarly, we can define the uniqueness of each function annotation term, phenotype, or disease, and then define the target function diversity *π*_*Target Fun*_ (i.e., effective number of target functions) of a TF or the function diversity *π*_*Fun*_ (i.e., effective number of known functions) of a gene.

## Additional files

Additional file 1: Supplementary Material. Supplementary Results, Methods, Figures (S1-S12), Tables (S1-S5, and S9). Tables S6, S7, S8, S10, S11 are available as separate files. Tables S10 and S11 correspond to the raw transcription factor–target gene (TFTG) relationships for 6000 and 20,000 windows, respectively, in GMT format [45, 47–49, 51–62, 71, 89, 90, 92–115, 119–121, 139–147, 155]. (DOCX 7176 kb)

Additional file 2: Table S6. A list of negatively associated functional concepts regulated by shared transcription factors. Negative association of two concepts is defined as a negative Phi coefficient defined based on the member genes of two functional concepts A and B. (XLSX 413 kb)

Additional file 3: Table S7. The complete transcription factor annotation results. –Log_10_ (*P* value) are provided in parentheses following the target functions. (XLSX 153 kb)

Additional file 4: Table S8. The complete list of TF pairs with significant target function overlaps but lower than expected target gene overlaps. Negative association (i.e., lower than expected target gene overlaps) of two TFs is defined as a negative Phi coefficient of the target gene overlaps of two TFs – TF1 and TF2. (XLSX 46 kb)

Additional file 5: Table S10. The raw transcription factor–target gene (TFTG) relationships in GMT file format for 6000bp window size. (GMT 987 kb)

Additional file 6: Table S11. The raw transcription factor–target gene (TFTG) relationships in GMT file format for 20,000bp window size. (GMT 1413 kb)

## Abbreviations

ChIP-Seq: chromatin immunoprecipitation sequencing
GO: gene ontology
PD: pharmacodynamics
PK: pharmacokinetic
TF: transcription factor
TG: target gene

## Footnotes

1 The target function association profile of a TF is comprised of the Pearson’s phi coefficients between the TF and all 3715 functional concepts. A lack of positive correlation between two profiles indicates that the two TFs are likely functionally unrelated based on the known functional concepts.

## References

[1] Whyte WA, Orlando DA, Hnisz D, Abraham BJ, Lin CY, Kagey MH (2013). Master transcription factors and mediator establish super-enhancers at key cell identity genes. Cell.

[2] Reik W (2007). Stability and flexibility of epigenetic gene regulation in mammalian development. Nature.

[3] López-Maury L, Marguerat S, Bähler J (2008). Tuning gene expression to changing environments: from rapid responses to evolutionary adaptation. Nat Rev Genet.

[4] Lenhard B, Sandelin A, Carninci P (2012). Regulatory elements: Metazoan promoters: emerging characteristics and insights into transcriptional regulation. Nature.

[5] Perissi V, Rosenfeld MG (2005). Controlling nuclear receptors: the circular logic of cofactor cycles. Nat Rev Mol Cell Biol.

[6] Maniatis T, Goodbourn S, Fischer J (1987). Regulation of inducible and tissue-specific gene expression. Science.

[7] Takahashi K, Yamanaka S (2006). Induction of pluripotent stem cells from mouse embryonic and adult fibroblast cultures by defined factors. Cell.

[8] Park I-H, Zhao R, West JA, Yabuuchi A, Huo H, Ince TA (2008). Reprogramming of human somatic cells to pluripotency with defined factors. Nature.

[9] Lee TI, Young RA (2013). Transcriptional regulation and its misregulation in disease. Cell.

[10] Jopling C, Boue S, Izpisua Belmonte JC (2011). Dedifferentiation, transdifferentiation and reprogramming: three routes to regeneration. Nat Rev Mol Cell Biol.

[11] Yamashita H, Takenoshita M, Sakurai M, Bruick RK, Henzel WJ, Shillinglaw W (2001). A glucose-responsive transcription factor that regulates carbohydrate metabolism in the liver. Proc Natl Acad Sci U S A.

[12] Wang R, Dillon CP, Shi LZ, Milasta S, Carter R, Finkelstein D (2011). The transcription factor myc controls metabolic reprogramming upon T lymphocyte activation. Immunity.

[13] Kaspar JW, Niture SK, Jaiswal AK (2009). Nrf2:INrf2 (Keap1) signaling in oxidative stress. Free Radic Biol Med.

[14] Tothova Z, Kollipara R, Huntly BJ, Lee BH, Castrillon DH, Cullen DE (2007). FoxOs are critical mediators of hematopoietic stem cell resistance to physiologic oxidative stress. Cell.

[15] Kumar A, Boriek AM (2003). Mechanical stress activates the nuclear factor-kappaB pathway in skeletal muscle fibers: a possible role in Duchenne muscular dystrophy. FASEB J.

[16] Mendez MG, Janmey PA (2012). Transcription factor regulation by mechanical stress. Int J Biochem Cell Biol.

[17] Whitney O, Pfenning AR, Howard JT, Blatti CA, Liu F, Ward JM (2014). Core and region-enriched networks of behaviorally regulated genes and the singing genome. Science.

[18] Pfenning AR, Hara E, Whitney O, Rivas MV, Wang R, Roulhac PL (2014). Convergent transcriptional specializations in the brains of humans and song-learning birds. Science.

[19] Greer EL, Brunet A (2005). FOXO transcription factors at the interface between longevity and tumor suppression. Oncogene.

[20] Salih DA, Brunet A (2008). FoxO transcription factors in the maintenance of cellular homeostasis during aging. Curr Opin Cell Biol.

[21] Tilstra J, Robinson A, Wang J (2012). NF-κB inhibition delays DNA damage–induced senescence and aging in mice. J Clin Invest.

[22] Yusuf D, Butland SL, Swanson MI, Bolotin E, Ticoll A, Cheung WA (2012). The transcription factor encyclopedia. Genome Biol.

[23] Vaquerizas JM, Kummerfeld SK, Teichmann SA, Luscombe NM (2009). A census of human transcription factors: function, expression and evolution. Nat Rev Genet.

[24] Ravasi T, Suzuki H, Cannistraci CV, Katayama S, Bajic VB, Tan K (2010). An atlas of combinatorial transcriptional regulation in mouse and man. Cell.

[25] Ashburner M, Ball CA, Blake JA, Botstein D, Butler H, Cherry JM (2000). Gene ontology: tool for the unification of biology. The Gene Ontology Consortium. Nat Genet.

[26] Kummerfeld SK, Teichmann SA (2006). DBD: a transcription factor prediction database. Nucleic Acids Res.

[27] The Gene Ontology Consortium (2015). Gene Ontology Consortium: going forward. Nucleic Acids Res.

[28] Yan J, Enge M, Whitington T, Dave K, Liu J, Sur I (2013). Transcription factor binding in human cells occurs in dense clusters formed around cohesin anchor sites. Cell.

[29] Weirauch MT, Yang A, Albu M, Cote AG, Montenegro-Montero A, Drewe P (2014). Determination and inference of eukaryotic transcription factor sequence specificity. Cell.

[30] Jolma A, Yin Y, Nitta KR, Dave K, Popov A, Taipale M (2015). DNA-dependent formation of transcription factor pairs alters their binding specificity. Nature.

[31] Furey TS (2012). ChIP–seq and beyond: new and improved methodologies to detect and characterize protein–DNA interactions. Nat Rev Genet.

[32] Gerstein MB, Kundaje A, Hariharan M, Landt SG, Yan K-K, Cheng C (2012). Architecture of the human regulatory network derived from ENCODE data. Nature.

[33] Kheradpour P, Kellis M (2014). Systematic discovery and characterization of regulatory motifs in ENCODE TF binding experiments. Nucleic Acids Res.

[34] Jolma A, Yan J, Whitington T, Toivonen J, Nitta KR, Rastas P (2013). DNA-binding specificities of human transcription factors. Cell.

[35] Cheng C, Yan K-K, Hwang W, Qian J, Bhardwaj N, Rozowsky J (2011). Construction and analysis of an integrated regulatory network derived from high-throughput sequencing data. PLoS Comput Biol.

[36] Neph S, Stergachis AB, Reynolds A, Sandstrom R, Borenstein E, Stamatoyannopoulos JA (2012). Circuitry and dynamics of human transcription factor regulatory networks. Cell.

[37] Bernstein BE, Birney E, Dunham I, Green ED, Gunter C, Snyder M (2012). An integrated encyclopedia of DNA elements in the human genome. Nature.

[38] Jiang P, Singh M (2014). CCAT: Combinatorial Code Analysis Tool for transcriptional regulation. Nucleic Acids Res.

[39] Ji H, Jiang H, Ma W, Wong WH. Using CisGenome to analyze ChIP-chip and ChIP-seq data. Curr Protoc Bioinformatics. 2011;Chapter 2:Unit2.13. 10.1002/0471250953.bi0213s33 PMC3072298 21400695

[40] Zhang Y, Liu T, Meyer CA, Eeckhoute J, Johnson DS, Bernstein BE (2008). Model-based analysis of ChIP-Seq (MACS). Genome Biol.

[41] Spyrou C, Stark R, Lynch AG, Tavaré S (2009). BayesPeak: Bayesian analysis of ChIP-seq data. BMC Bioinformatics.

[42] Zhu LJ, Gazin C, Lawson ND, Pagès H, Lin SM, Lapointe DS (2010). ChIPpeakAnno: a Bioconductor package to annotate ChIP-seq and ChIP-chip data. BMC Bioinformatics.

[43] McLean CY, Bristor D, Hiller M, Clarke SL, Schaar BT, Lowe CB (2010). GREAT improves functional interpretation of cis-regulatory regions. Nat Biotechnol.

[44] Welch RP, Lee C, Imbriano PM, Patil S, Weymouth TE, Smith RA (2014). ChIP-Enrich: Gene set enrichment testing for ChIP-seq data. Nucleic Acids Res.

[45] Cheng C, Min R, Gerstein M (2011). TIP: a probabilistic method for identifying transcription factor target genes from ChIP-seq binding profiles. Bioinformatics.

[46] Barabási A (1999). Emergence of Scaling in Random Networks. Science.

[47] Gude NA, Emmanuel G, Wu W, Cottage CT, Fischer K, Quijada P (2008). Activation of Notch-mediated protective signaling in the myocardium. Circ Res.

[48] Li Y, Hiroi Y, Ngoy S, Okamoto R, Noma K, Wang C-Y (2011). Notch1 in bone marrow-derived cells mediates cardiac repair after myocardial infarction. Circulation.

[49] Hamosh A, Scott AF, Amberger JS, Bocchini CA, McKusick VA (2005). Online Mendelian Inheritance in Man (OMIM), a knowledgebase of human genes and genetic disorders. Nucleic Acids Res.

[50] Phillips JD, Steensma DP, Pulsipher MA, Spangrude GJ, Kushner JP (2007). Congenital erythropoietic porphyria due to a mutation in GATA1: the first trans-acting mutation causative for a human porphyria. Blood.

[51] DeSandro A, Nagarajan UM, Boss JM (1999). The bare lymphocyte syndrome: molecular clues to the transcriptional regulation of major histocompatibility complex class II genes. Am J Hum Genet.

[52] Reith W, Mach B (2001). The bare lymphocyte syndrome and the regulation of MHC expression. Annu Rev Immunol.

[53] Masternak K, Barras E, Zufferey M, Conrad B, Corthals G, Aebersold R (1998). A gene encoding a novel RFX-associated transactivator is mutated in the majority of MHC class II deficiency patients. Nat Genet.

[54] Clausen BE, Waldburger JM, Schwenk F, Barras E, Mach B, Rajewsky K (1998). Residual MHC class II expression on mature dendritic cells and activated B cells in RFX5-deficient mice. Immunity.

[55] Sulem P, Gudbjartsson DF, Geller F, Prokopenko I, Feenstra B, Aben KKH (2011). Sequence variants at CYP1A1-CYP1A2 and AHR associate with coffee consumption. Hum Mol Genet.

[56] Cornelis MC, Monda KL, Yu K, Paynter N, Azzato EM, Bennett SN (2011). Genome-wide meta-analysis identifies regions on 7p21 (AHR) and 15q24 (CYP1A2) as determinants of habitual caffeine consumption. PLoS Genet.

[57] Cornelis MC, Byrne EM, Esko T, Nalls MA, Ganna A, Paynter N (2015). Genome-wide meta-analysis identifies six novel loci associated with habitual coffee consumption. Mol Psychiatry.

[58] Reiner AP, Gross MD, Carlson CS, Bielinski SJ, Lange LA, Fornage M (2009). Common coding variants of the HNF1A gene are associated with multiple cardiovascular risk phenotypes in community-based samples of younger and older European-American adults: the Coronary Artery Risk Development in Young Adults Study and The Cardiovascular Health Study. Circ Cardiovasc Genet.

[59] Steele AM, Shields BM, Shepherd M, Ellard S, Hattersley AT, Pearson ER (2010). Increased all-cause and cardiovascular mortality in monogenic diabetes as a result of mutations in the HNF1A gene. Diabet Med.

[60] Pontoglio M, Barra J, Hadchouel M, Doyen A, Kress C, Bach JP (1996). Hepatocyte nuclear factor 1 inactivation results in hepatic dysfunction, phenylketonuria, and renal Fanconi syndrome. Cell.

[61] Jostins L, Ripke S, Weersma RK, Duerr RH, McGovern DP, Hui KY (2012). Host-microbe interactions have shaped the genetic architecture of inflammatory bowel disease. Nature.

[62] Shimano H, Shimomura I, Hammer RE, Herz J, Goldstein JL, Brown MS (1997). Elevated levels of SREBP-2 and cholesterol synthesis in livers of mice homozygous for a targeted disruption of the SREBP-1 gene. J Clin Invest.

[63] Baeuerle PA, Baichwal VR (1997). NF-kB as a frequent target for immunosuppressive and anti-inflammatory molecules. Adv Immunol.

[64] Masternak K, Muhlethaler-Mottet A, Villard J, Zufferey M, Steimle V, Reith W (2000). CIITA is a transcriptional coactivator that is recruited to MHC class II promoters by multiple synergistic interactions with an enhanceosome complex. Genes Dev.

[65] Fliegauf M, L Bryant V, Frede N, Slade C, Woon S-T, Lehnert K (2015). Haploinsufficiency of the NF-κB1 subunit p50 in common variable immunodeficiency. Am J Hum Genet.

[66] Sogawa K, Fujii-Kuriyama Y (1997). Ah receptor, a novel ligand-activated transcription factor. J Biochem.

[67] Lamba J, Lamba V, Schuetz E (2005). Genetic variants of PXR (NR1I2) and CAR (NR1I3) and their implications in drug metabolism and pharmacogenetics. Curr Drug Metab.

[68] Ma Q (2008). Xenobiotic-activated receptors: from transcription to drug metabolism to disease. Chem Res Toxicol.

[69] Li L, Davie JR (2010). The role of Sp1 and Sp3 in normal and cancer cell biology. Ann Anat.

[70] Hollstein M, Sidransky D, Vogelstein B, Harris C (1991). p53 mutations in human cancers. Science.

[71] Teslovich TM, Musunuru K, Smith AV, Edmondson AC, Stylianou IM, Koseki M (2010). Biological, clinical and population relevance of 95 loci for blood lipids. Nature.

[72] Ren B, Cam H, Takahashi Y, Volkert T, Terragni J, Young RA (2002). E2F integrates cell cycle progression with DNA repair, replication, and G(2)/M checkpoints. Genes Dev.

[73] Gaubatz S, Lindeman GJ, Ishida S, Jakoi L, Nevins JR, Livingston DM (2000). E2F4 and E2F5 play an essential role in pocket protein-mediated G1 control. Mol Cell.

[74] Griffon A, Barbier Q, Dalino J, van Helden J, Spicuglia S, Ballester B (2015). Integrative analysis of public ChIP-seq experiments reveals a complex multi-cell regulatory landscape. Nucleic Acids Res.

[75] Bunger MK, Wilsbacher LD, Moran SM, Clendenin C, Radcliffe LA, Hogenesch JB (2000). Mop3 is an essential component of the master circadian pacemaker in mammals. Cell.

[76] Storch K-F, Paz C, Signorovitch J, Raviola E, Pawlyk B, Li T (2007). Intrinsic circadian clock of the mammalian retina: importance for retinal processing of visual information. Cell.

[77] Rossner MJ, Oster H, Wichert SP, Reinecke L, Wehr MC, Reinecke J (2008). Disturbed clockwork resetting in Sharp-1 and Sharp-2 single and double mutant mice. PLoS One.

[78] Honma S, Kawamoto T, Takagi Y, Fujimoto K, Sato F, Noshiro M (2002). Dec1 and Dec2 are regulators of the mammalian molecular clock. Nature.

[79] Zambon C, Pengo V, Padrini R, Basso D, Schiavon S, Fogar P (2011). Research article algorithm for warfarin dosing: an Italian retrospective study research article. Pharmacogenomics.

[80] Wietholtz H, Zysset T, Kreiten K, Kohl D, Büchsel R, Matern S (1989). Effect of phenytoin, carbamazepine, and valproic acid on caffeine metabolism. Eur J Clin Pharmacol.

[81] Enga KF, Braekkan SK, Hansen-Krone IJ, Wilsgaard T, Hansen J-B (2011). Coffee consumption and the risk of venous thromboembolism: the Tromsø study. J Thromb Haemost.

[82] Li J, Seibold P, Chang-Claude J, Flesch-Janys D, Liu J, Czene K (2011). Coffee consumption modifies risk of estrogen-receptor negative breast cancer. Breast Cancer Res.

[83] Lowcock EC, Cotterchio M, Anderson LN, Boucher BA, El-Sohemy A (2013). High coffee intake, but not caffeine, is associated with reduced estrogen receptor negative and postmenopausal breast cancer risk with no effect modification by CYP1A2 genotype. Nutr Cancer.

[84] Nkondjock A, Ghadirian P, Kotsopoulos J, Lubinski J, Lynch H, Kim-Sing C (2006). Coffee consumption and breast cancer risk among BRCA1 and BRCA2 mutation carriers. Int J Cancer.

[85] Ascherio A, Chen H, Schwarzschild MA, Zhang SM, Colditz GA, Speizer FE (2003). Caffeine, postmenopausal estrogen, and risk of Parkinson’s disease. Neurology.

[86] Ascherio A, Weisskopf MG, O’Reilly EJ, McCullough ML, Calle EE, Rodriguez C (2004). Coffee consumption, gender, and Parkinson’s disease mortality in the cancer prevention study II cohort: the modifying effects of estrogen. Am J Epidemiol.

[87] Nagata C, Kabuto M, Shimizu H (1998). Association of coffee, green tea, and caffeine intakes with serum concentrations of estradiol and sex hormone-binding globulin in premenopausal Japanese women. Nutr Cancer.

[88] Page L, Brin S, Motwani R, Winograd T. The PageRank Citation Ranking: Bringing Order to the Web. Stanford InfoLab. 1999. http://ilpubs.stanford.edu:8090/422/. Accessed 19 Dec 2017.

[89] Yu H, Gerstein M (2006). Genomic analysis of the hierarchical structure of regulatory networks. Proc Natl Acad Sci U S A.

[90] Bhardwaj N, Yan K-K, Gerstein MB (2010). Analysis of diverse regulatory networks in a hierarchical context shows consistent tendencies for collaboration in the middle levels. Proc Natl Acad Sci U S A.

[91] McCall MN, Uppal K, Jaffee HA, Zilliox MJ, Irizarry RA (2011). The gene expression barcode: Leveraging public data repositories to begin cataloging the human and murine transcriptomes. Nucleic Acids Res.

[92] Futreal PA, Coin L, Marshall M, Down T, Hubbard T, Wooster R (2004). A census of human cancer genes. Nat Rev Cancer.

[93] Rebouissou S, Vasiliu V, Thomas C, Bellanné-Chantelot C, Bui H, Chrétien Y (2005). Germline hepatocyte nuclear factor 1alpha and 1beta mutations in renal cell carcinomas. Hum Mol Genet.

[94] Yamada S, Nishigori H, Onda H, Utsugi T, Yanagawa T, Maruyama T (1997). Identification of mutations in the hepatocyte nuclear factor (HNF)-1 alpha gene in Japanese subjects with IDDM. Diabetes.

[95] Hegele RA, Cao H, Harris SB, Hanley AJ, Zinman B (1999). The hepatic nuclear factor-1alpha G319S variant is associated with early-onset type 2 diabetes in Canadian Oji-Cree. J Clin Endocrinol Metab.

[96] Kathiresan S, Manning AK, Demissie S, D’Agostino RB, Surti A, Guiducci C (2007). A genome-wide association study for blood lipid phenotypes in the Framingham Heart Study. BMC Med Genet.

[97] Mandeville I, Aubin J, LeBlanc M, Lalancette-Hébert M, Janelle M-F, Tremblay GM (2006). Impact of the loss of Hoxa5 function on lung alveogenesis. Am J Pathol.

[98] Xiao X, Zuo X, Davis AA, McMillan DR, Curry BB, Richardson JA (1999). HSF1 is required for extra-embryonic development, postnatal growth and protection during inflammatory responses in mice. EMBO J.

[99] Neve B, Fernandez-Zapico ME, Ashkenazi-Katalan V, Dina C, Hamid YH, Joly E (2005). Role of transcription factor KLF11 and its diabetes-associated gene variants in pancreatic beta cell function. Proc Natl Acad Sci U S A.

[100] Collins S, Groudine M (1982). Amplification of endogenous myc-related DNA sequences in a human myeloid leukaemia cell line. Nature.

[101] Yokota J, Tsunetsugu-Yokota Y, Battifora H, Le Fevre C, Cline M (1986). Alterations of myc, myb, and rasHa proto-oncogenes in cancers are frequent and show clinical correlation. Science.

[102] Macfarlane WM, Frayling TM, Ellard S, Evans JC, Allen LI, Bulman MP (1999). Missense mutations in the insulin promoter factor-1 gene predispose to type 2 diabetes. J Clin Invest.

[103] Hani EH, Stoffers DA, Chèvre JC, Durand E, Stanojevic V, Dina C (1999). Defective mutations in the insulin promoter factor-1 (IPF-1) gene in late-onset type 2 diabetes mellitus. J Clin Invest.

[104] Coppola E, Rallu M, Richard J, Dufour S, Riethmacher D, Guillemot F (2010). Epibranchial ganglia orchestrate the development of the cranial neurogenic crest. Proc Natl Acad Sci U S A.

[105] Vohl MC, Lepage P, Gaudet D, Brewer CG, Bétard C, Perron P (2000). Molecular scanning of the human PPARa gene: association of the L162v mutation with hyperapobetalipoproteinemia. J Lipid Res.

[106] Gross B, Hennuyer N, Bouchaert E, Rommens C, Grillot D, Mezdour H (2011). Generation and characterization of a humanized PPARδ mouse model. Br J Pharmacol.

[107] Mao C-A, Tsai W-W, Cho J-H, Pan P, Barton MC, Klein WH (2011). Neuronal transcriptional repressor REST suppresses an Atoh7-independent program for initiating retinal ganglion cell development. Dev Biol.

[108] Liang G, Yang J, Horton JD, Hammer RE, Goldstein JL, Brown MS (2002). Diminished hepatic response to fasting/refeeding and liver X receptor agonists in mice with selective deficiency of sterol regulatory element-binding protein-1c. J Biol Chem.

[109] Lin AE, Semina EV, Daack-Hirsch S, Roeder ER, Curry CJ, Rosenbaum K (2000). Exclusion of the branchio-oto-renal syndrome locus (EYA1) from patients with branchio-oculo-facial syndrome. Am J Med Genet.

[110] Milunsky JM, Maher TA, Zhao G, Roberts AE, Stalker HJ, Zori RT (2008). TFAP2A mutations result in branchio-oculo-facial syndrome. Am J Hum Genet.

[111] Gestri G, Osborne RJ, Wyatt AW, Gerrelli D, Gribble S, Stewart H (2009). Reduced TFAP2A function causes variable optic fissure closure and retinal defects and sensitizes eye development to mutations in other morphogenetic regulators. Hum Genet.

[112] Chen PL, Chen YM, Bookstein R, Lee WH (1990). Genetic mechanisms of tumor suppression by the human p53 gene. Science.

[113] Halevy O, Michalovitz D, Oren M (1990). Different tumor-derived p53 mutants exhibit distinct biological activities. Science.

[114] Chiang YJ, Difilippantonio MJ, Tessarollo L, Morse HC, Hodes RJ (2012). Exon 1 disruption alters tissue-specific expression of mouse p53 and results in selective development of B cell lymphomas. PLoS One.

[115] Pelletier J, Bruening W, Li FP, Haber DA, Glaser T, Housman DE (1991). WT1 mutations contribute to abnormal genital system development and hereditary Wilms’ tumour. Nature.

[116] Wang J, Zhuang J, Iyer S, Lin X-Y, Greven MC, Kim B-H (2013). Factorbook.org: a Wiki-based database for transcription factor-binding data generated by the ENCODE consortium. Nucleic Acids Res.

[117] Bailey T, Krajewski P, Ladunga I, Lefebvre C, Li Q, Liu T (2013). Practical guidelines for the comprehensive analysis of ChIP-seq data. PLoS Comput Biol.

[118] Barrett T, Wilhite SE, Ledoux P, Evangelista C, Kim IF, Tomashevsky M (2013). NCBI GEO: archive for functional genomics data sets--update. Nucleic Acids Res.

[119] Karolchik D (2003). The UCSC Genome Browser Database. Nucleic Acids Res.

[120] Storey JD (2003). The positive false discovery rate: a Bayesian interpretation and the q-value. Ann Stat.

[121] Storey JD, Tibshirani R (2003). Statistical significance for genomewide studies. Proc Natl Acad Sci U S A.

[122] Bader GD (2003). BIND: the Biomolecular Interaction Network Database. Nucleic Acids Res.

[123] Bovolenta LA, Acencio ML, Lemke N (2012). HTRIdb: an open-access database for experimentally verified human transcriptional regulation interactions. BMC Genomics.

[124] Portales-Casamar E, Arenillas D, Lim J, Swanson MI, Jiang S, McCallum A (2009). The PAZAR database of gene regulatory information coupled to the ORCA toolkit for the study of regulatory sequences. Nucleic Acids Res.

[125] Jiang C, Xuan Z, Zhao F, Zhang MQ (2007). TRED: a transcriptional regulatory element database, new entries and other development. Nucleic Acids Res.

[126] Yang VW (1998). Issues and opinions in nutrition. Eukaryotic transcription factors: identification, characterization. J Nutr.

[127] Geertz M, Maerkl SJ (2010). Experimental strategies for studying transcription factor-DNA binding specificities. Brief Funct Genomics.

[128] Joshi-Tope G, Gillespie M, Vastrik I, D’Eustachio P, Schmidt E, de Bono B (2005). Reactome: a knowledgebase of biological pathways. Nucleic Acids Res.

[129] Liberzon A, Subramanian A, Pinchback R, Thorvaldsdóttir H, Tamayo P, Mesirov JP (2011). Molecular signatures database (MSigDB) 3.0.. Bioinformatics.

[130] Hewett M (2002). PharmGKB: the Pharmacogenetics Knowledge Base. Nucleic Acids Res.

[131] Mailman MD, Feolo M, Jin Y, Kimura M, Tryka K, Bagoutdinov R (2007). The NCBI dbGaP database of genotypes and phenotypes. Nat Genet.

[132] Welter D, MacArthur J, Morales J, Burdett T, Hall P, Junkins H (2014). The NHGRI GWAS Catalog, a curated resource of SNP-trait associations. Nucleic Acids Res.

[133] Schriml LM, Arze C, Nadendla S, Chang Y-WW, Mazaitis M, Felix V (2012). Disease Ontology: a backbone for disease semantic integration. Nucleic Acids Res.

[134] Maglott D, Ostell J, Pruitt KD, Tatusova T (2005). Entrez Gene: gene-centered information at NCBI. Nucleic Acids Res.

[135] Kanehisa M (2000). KEGG: Kyoto Encyclopedia of Genes and Genomes. Nucleic Acids Res.

[136] Nishimura D (2001). BioCarta. Biotech Softw Internet Rep.

[137] Mehta CR (1986). Algorithm 643. FEXACT: a FORTRAN subroutine for Fisher’s exact test on unordered rxc contingency tables. ACM Trans Math Softw.

[138] Benjamini Y, Hochberg Y (1995). Controlling the false discovery rate: a practical and powerful approach to multiple testing. J R Stat Soc Ser B.

[139] Hani EH, Suaud L, Boutin P, Chèvre JC, Durand E, Philippi A (1998). A missense mutation in hepatocyte nuclear factor-4 alpha, resulting in a reduced transactivation activity, in human late-onset non-insulin-dependent diabetes mellitus. J Clin Invest.

[140] Wang H, Maechler P, Antinozzi PA, Hagenfeldt KA, Wollheim CB (2000). Hepatocyte nuclear factor 4alpha regulates the expression of pancreatic beta -cell genes implicated in glucose metabolism and nutrient-induced insulin secretion. J Biol Chem.

[141] Pingault V, Bondurand N, Kuhlbrodt K, Goerich DE, Préhu MO, Puliti A (1998). SOX10 mutations in patients with Waardenburg-Hirschsprung disease. Nat Genet.

[142] Hildebrandt F, Benzing T, Katsanis N (2011). Ciliopathies. N Engl J Med.

[143] Forsythe E, Beales PL (2013). Bardet-Biedl syndrome. Eur J Hum Genet.

[144] Bisgrove BW, Makova S, Yost HJ, Brueckner M (2012). RFX2 is essential in the ciliated organ of asymmetry and an RFX2 transgene identifies a population of ciliated cells sufficient for fluid flow. Dev Biol.

[145] Chung M-I, Peyrot SM, LeBoeuf S, Park TJ, McGary KL, Marcotte EM (2012). RFX2 is broadly required for ciliogenesis during vertebrate development. Dev Biol.

[146] Brown MS, Goldstein JL (1997). The SREBP Pathway: regulation of cholesterol metabolism by proteolysis of a membrane-bound transcription factor. Cell.

[147] Hua X, Nohturfft A, Goldstein JL, Brown MS (1996). Sterol resistance in CHO cells traced to point mutation in SREBP cleavage-activating protein. Cell.

[148] Smoot ME, Ono K, Ruscheinski J, Wang P-L, Ideker T (2011). Cytoscape 2.8: new features for data integration and network visualization. Bioinformatics.

[149] Hildebrand JD, Soriano P (2002). Overlapping and unique roles for C-terminal binding protein 1 (CtBP1) and CtBP2 during mouse development. Mol Cell Biol.

[150] Seth A, Watson DK (2005). ETS transcription factors and their emerging roles in human cancer. Eur J Cancer.

[151] He A, Ma Q, Cao J, von Gise A, Zhou P, Xie H (2012). Polycomb repressive complex 2 regulates normal development of the mouse heart. Circ Res.

[152] Malkin D, Li F, Strong L, Fraumeni J, Nelson C, Kim D (1990). Germ line p53 mutations in a familial syndrome of breast cancer, sarcomas, and other neoplasms. Science.

[153] Coon H, Xin Y, Hopkins PN, Cawthon RM, Hasstedt SJ, Hunt SC (2005). Upstream stimulatory factor 1 associated with familial combined hyperlipidemia, LDL cholesterol, and triglycerides. Hum Genet.

[154] Pajukanta P, Lilja HE, Sinsheimer JS, Cantor RM, Lusis AJ, Gentile M (2004). Familial combined hyperlipidemia is associated with upstream transcription factor 1 (USF1). Nat Genet.

[155] Fernando MMA, Stevens CR, Walsh EC, De Jager PL, Goyette P, Plenge RM, Vyse TJ, Rioux JD, Fisher EMC. Defining the Role of the MHC in Autoimmunity: A Review and Pooled Analysis. PLoS Genetics. 2008;4(4):e1000024. 10.1371/journal.pgen.1000024 PMC2291482 18437207

