# Supplementary material for: Systematic target function annotation of human transcription factors
Source: BMC Biol. 2018 Jan 10;16:4. doi: 10.1186/s12915-017-0469-0 (PMC5795274; doi:10.1186/s12915-017-0469-0)
Supplement: Supplementary file 1 — Supplementary Results, Methods, Figures (S1-S12), Tables (S1-S5, and S9). Tables S6, S7, S8, S10, S11 are available as separate files. Tables S10 and S11 correspond to the raw transcription factor–target gene (TFTG) relationships for 6000 and 20,000 windows, respectively, in GMT format [45, 47–49, 51–62, 71, 89, 90, 92–115, 119–121, 139–147, 155]. (DOCX 7176 kb) [file 12915_2017_469_MOESM1_ESM.docx]

**Supplementary Text**

**Systematic Target Function Annotation of Human Transcription Factors**

Yong Fuga Li^1, 2, 3^, Russ B. Altman^2, 4^

Affiliations:

^1^Stanford Genome Technology Center, Stanford, California, USA

^2^Department of Bioengineering, Stanford University, Stanford, California, USA

^3^Current affiliation: Department of Bioinformatics, Illumina Inc., San Diego, USA

^4^Department of Genetics, Stanford University, Stanford, California, USA

**1. Supplemental Results**

**1.1. Negative Associations between Transcription Factors and Functions**

Overall, 279 (73%) transcription factors are annotated by at least one functional term, while around 27% (104) transcription factors are annotated at least once by negatively associated gene functions or phenotypes. Percentage of negative TF association is lowest for pharmacogenomic pathways (2%) and Mendelian diseases (5%), and highest for GWAS phenotypes (51%) and gene molecular functions (40%). Many of the negative associations may simply indicate that the TFs and the function are irrelevant, although some of the negative TF-phenotype associations appear to be biologically meaningful. For example, NOTCH1 is negatively associated with myocardial infarction (OR = 0.47, Hypergeometric test p-value = 3.8 × 10^-7^), while NOTCH1 is known to mediate cardiac repair following myocardial infarction (47, 48).

**1.2. Transcription Factors have Preferences on the Type of Function Annotations**

We observed extensive TF sharing among the 6 types of gene functions (**Fig. S5A**).

Meanwhile, we observed systematic preference of different types of TFs to different sources of gene functions. For example, HNF1A target functions are 53 fold enriched for PK/PD pathways, 23 fold enriched for GWAS phenotypes, and 4 fold enriched for Mendelian diseases, while TCF12 target functions are 16 fold enriched for GO molecular functions, and 4 fold enriched for Reactome pathways. In total, there are 62 TFs with significant biases toward specific types of functions (**Fig. S5B)**. We note that different types of gene annotations are focused on different levels or different aspects of functions in living organisms. The biases of TFs toward specific types of functions suggest some high-level architecture of the TF-target function network. Specifically, different TFs may be in charge of functions of different levels from molecular to phenotype.

**1.3. Transcription Factors Sharing Reveals Redundant or Related Functions**

From the TF-target function relationships, we observed extensive regulator sharing among the functional concepts (e.g. *Cor pulmonale* and primary pulmonary hypertension) as well as target function overlaps among transcription factors (e.g. CTBP2 and SUZ12 in **Fig. 3B** top left). Redundancies or associations among functional concepts from both same sources and different annotation sources are observed (**Fig. 3B**). One cause of the concept similarities is the built-in redundancy of functional concepts within sources such as Gene Ontology, which defines concepts hierarchically, and between sources such as the GO biological processes and Reactome pathways, both of which covers signaling pathways. Another cause is the inherent biological relatedness of apparently different concepts, such as *beta-cell development* and *diabetes*, both of which are identified as targets of KLF11 and PDX1 (**Fig. 3B** bottom left).

We quantified the redundancy between functional concepts by their member gene overlap using Pearson’s $\phi$ coefficient, and estimated the total effective number of functional concepts to be 1316 compared to the total of 3715. Relatedly, 954 pairs of functional concepts are regulated by identical set of TFs, and 141 pairs of concepts have identical member genes. GO biological processes have the highest redundancy of 3.7 fold, while the GWAS phenotypes have the lowest redundancies of 1.2 fold (**Table S2**).

To further reveal the overall concept relatedness/redundancy between different sources of functional concepts, we calculated the average number of shared TFs per pairs of functional concepts from the 6 different sources (**Fig. S5B**). We find molecular functions, biological processes, and Reactome pathways cluster closely with >0.5 TFs shared per pair of functions from different sources, compared to 0.6-1.3 TFs shared per pair of functions from same sources). Pharmacogenomic pathways and Mendelian diseases form another cluster with 0.08 TFs shared per pair of functions from different sources, compared to 0.16 or 0.22 TFs shared per pair of functions from same sources.

**1.4. Basic Statistics of Functional Diversity and Regulator Diversity of TFs**

Overall, the average uniqueness of all functional concepts is only 0.4 (i.e. average degeneracy 2.8). There are on average 23.9 target functions per TF, while the target function diversity is only 3.7 with a degeneracy of around 6.1 fold, higher than the average degeneracy of all functions. On the other hand, the transcription factors are also highly related to each other, with an average uniqueness of 0.6, and effectively 226.0 functionally unique TFs out of 384. For each TF, there are 11.8 upstream regulators, while the regulator diversity is 5.2 and regulator degeneracy 2.2. **Fig. 6A** give an example of TF HNF1A, which have 6.3 effective target functions and 3.8 effective regulators (see **Fig. S11** for more transcription factors RXF5 and NFKB1; and also examples of non-TF gene MTHFR).

**1.5. Removing the impact of human research biases on the association between TF regulator diversity and function diversity**

The TFTG compendium contains TF-target gene relationships from the literature of low throughput studies. Such data are prone to human biases toward the perceived important TFs. These popular TFs can have more target genes as well as upstream regulators reported due to higher research efforts, hence creating an artificial association between the number of regulators and number of identified target functions. We corrected such biases by including the number of target genes of TF as a confounding variable (see main text). As a stricter validation, we completely removed the low-throughput data in the TFTG data, retained only the target genes for 262 TFs from ChIP-seq studies, and redid all analyses. The selection of TFs in ChIP-seq experiment remains biased toward the well-known TFs. Popular TFs however should no longer have more upstream TFs due to preferred study of the regulators of these TFs. Despite a smaller number of TFs and a loss of the most reliable target genes for the remaining TFs, regulator diversity and function diversity remain significantly associated (p-value 0.015, or 0.0086 when controlling for the number of target genes).

**1.6. Hierarchical location of a TF in the regulatory network is not associated with multifunctionality of the TF**

Aside from regulation diversity, we discover that the hierarchical seniority of a TF, as measured by the PageRank of a TF in the core TRTG network, is also associated with the (known) function diversity (p-value 0.0023, PCC 0.15) and the regulator diversity of a TF (p-value 0.025, PCC 0.12). There is an even stronger association between PageRank and the target function diversity of a TF (p-value 1.2e-42 and PCC 0.62). These indicate that a transcription factor at the top of the regulatory hierarchy tends to have more diverse functions. This correlation however may be caused by the shared association of PageRank and the target-function diversity with the number of target genes of a TF, as they are both defined on the target genes. Indeed, after controlling for the target gene size through a linear model, neither the PageRank-function diversity or PageRank-target function diversity associations are significant (p-values 0.94 and 0.051 respectively). Similar behavior is observed for other TF hierarchical rank measures defined based on breadth first search (BFS) and percentage of regulatory target genes (89, 90).

**1.7. Gene’s Regulator Diversity and Multifunctionality**

Significant associations between function and regulator diversities of genes are observed for specific types of functional annotations (**Supplemental Fig. S10**), including signaling/metabolic pathways (p-value 1.5 × 10^-6^), biological processes (p-value 1.5 × 10^-6^), and GWAS phenotypes (p-value 1.1 × 10^-10^), while the diversity of GWAS phenotype of a gene is inversely correlated with its regulator diversity. Genes regulated by 10 or more other TFs are 14% less likely to be associated with any GWAS phenotypes, and 46% less likely to be associated with 5 or more phenotypes. Note that we used the Coding gene universe when analyzing GWAS phenotype associations, and the Literature Rich gene universe when analyzing other annotations. Both of these are conservative choices of gene universes. Using Literature Rich gene universe will lead to more significant negative association between TF regulation and phenotypes, while using Coding gene universe will lead to more significant positive associations between TF regulation and signaling & metabolic pathways, biological processes, and molecular functions.

We removed the low-throughput data in the TFTG data, and only retained the target genes for 262 TFs from ChIP-seq studies. The associations of TFs’ regulator diversity with specific types of functions are PCC 0.17, p-value 0.007 for GWAS phenotypes; PCC -0.0063, p-value 0.92 for Mendelian disease; PCC 0.11 p-value 0.093 for PK & PD pathways; PCC 0.086, p-value 0.17 for molecular functions, PCC 0.14, p-value 0.032 for biological processes; PCC 0.15, p-value 0.015 for signaling & metabolic pathways. Meanwhile, for general genes (TFs and non-TFs), there is still a significant positive association between regulator diversity and signaling/metabolic pathway diversity (PCC 0.072, p-value 1.3e-6), and a significant negative association for GWAS phenotypes (PCC -0.11, p-value 6.8 e-19).

For the 10792 general genes that have regulators, function annotations as well as expression data, genes’ regulator diversity and expression diversity are correlated (Spearman rank correlation 0.22, p-value 3.7 × 10^-120^ for diversities, or correlation 0.26, p-value 1.0 × 10^-165^ for the raw counts), and genes’ regulator diversity and function diversity are also correlated (Spearman rank correlation 0.12, p-value 1.0 × 10^-33^).

**1.8. Examples of Mendelian Diseases Targeted by Transcription Factors**

We identified hereditary mitochondrial metabolism disease as a target disease of HNF4A, while it has been reported that HNF4A is mutated in hereditary noninsulin-dependent diabetes mellitus (NIDDM) (139), which is a mitochondrial disease. HNF4A is also mutated in maturity-onset diabetes of the young (MODY1) (140), and the MODY1 mutant form of HNF4A is known to cause mitochondrial function defects as well as impairment of nutrient-stimulated insulin release. Transcription factor SOX10 is predicted to be associated with Waardenburg's syndrome (**Table 1**) as well as Hirschsprung's disease and megacolon (not shown). Notably, SOX10 is a known causal gene of Waardenburg's syndrome type 4C and Hirschsprung’s disease (141), while megacolon is a symptom of Hirschsprung’s disease. Another example is the association of RFX2 with Bardet-Biedl syndrome (BBS), which is a pleiotropic recessive genetic disorder belonging to a group of diseases called ciliopathy (142). There are at least sixteen known BBS causing genes (or nineteen according to OMIM) covering around 80% of the diagnosed cases (143). These BBS causing genes encode proteins involved in cilia biogenesis and function. Transcription factor RFX2 is not known to mutate in BBS patients, however, RFX2 together with RFX3 and RFX4, are key regulators of cilia genesis in mouse and other animal models (144, 145).

In addition to above examples for GATA1, HNF4A, SOX10 and RFX2, other target Mendelian diseases among the top twenty are directly supported by human or mouse phenotypes upon direct mutation of the TFs. They are heart septal defect and congenital heart diseases for CTBP2, heart septal defect for SUZ12 (**Fig. 3C**), cancers for ETS1 and TP53, and inherited metabolic disorder for USF1.

**1.9. Examples of Complex Phenotypes Targeted by Transcription Factors**

RFX5 mutations cause Bare lymphocyte syndrome II through impaired MHC II protein expression (146, 147), while MHC II is genetically linked to multiple autoimmune diseases (148). SREBF1 (sterol regulatory element binding transcription factor 1) is identified to target blood LDL (low density lipoprotein) level (p-value 2.2 × 10^-6^), and not surprisingly, SREBF1 is a known regulator of LDL proteins and genes involved in sterol synthesis (62, 149). Consistently, SREBF1 is associated with the statin pharmacodynamics pathway (PharmGKB) and multiple biological processes and pathways related to lipid metabolism. The SREBF1 LDL association is also directly supported by mouse mutation phenotypes (62, 150).

Habitual coffee consumption is a significant target phenotype of AHR (Aryl hydrocarbon receptor, **Fig. 3E**), while *AHR* gene locus itself is also strongly associated with coffee consumption and habitual coffee consumption in GWAS studies (55–57). HNF1A (**Fig. 3G**) targets three phenotypes, including the serum cell-free DNA level as an indicator of cardiovascular disease risk, serum bilirubin levels as a measure of cholelithiasis risk, and F-cell levels as an indicator of sickle-cell anemia. The first two phenotypes are validated by genetic evidence. HNF1A mutation is associated with elevated risk of cardiovascular diseases in diabetes patients (59) while SNPs of HNF1A is found to be associated with cardiovascular disease risks in young and old European Americans (58). The HNF1A-cholelithiasis connection is supported by HNF1A knockout mouse (60), which showed elevated blood bilirubin levels and jaundice. Consistently, HNF1A also targets Reactome pathway *synthesis of bile acids and bile salts* (**Fig. 3G**).

**2. Supplemental Methods**

**2.1. Modified TIP algorithm for Exacting Target Genes from ChIP-Seq Data**

The binding signals from target genes were differentiated from that from non-target genes using a modified version of the TIP algorithm (45). Specifically, the modified implementation accepts both fixed step and variable step wiggle formatted files as well as other commonly used genome feature formats, such as bed and bigWig. It properly handles the 0-based start and 1-based end coordinates of transcript annotations and the 1-based coordinates in wig files. Genomic regions with read density less than 1 read per million total reads are ignored. In addition, it only evaluates the unambiguously mapped transcripts, and removes redundant promoter regions (i.e. transcripts with identical TSS are merged). RefSeq gene annotations on human genome hg18 and hg19 were obtained from the UCSC genome browser (<https://genome.ucsc.edu>) (119). Instead of the dot product scoring functions used in TIP, we used log likelihood ratio to achieve a Gaussian like distribution of the scores,

$$S_{g}=\sum_{l=-3000}^{3000} x_{gl}\cdot\log\left( \frac{m_{l.gene}}{m_{l.null}} \right)\text{,}$$

where $x_{gl}$ is the read counts at location $l$ within the promoter region, and score $S_{g}$ for promoter/gene $g$ is calculated as the read-counts weighted sum of the log likelihood ratio between the average signal distribution $m_{l.gene}$ and the even distribution $m_{l.null}$. Both 6K and 20K window sizes are evaluated, and the 6k window is used in the end spanning ±3K base pairs 5’ and 3’ to the TSS. P-values are then computed by assuming a Gaussian distribution of $S_{g}$. The target genes of each TF in each experiment are then obtained at q-value cutoff of 0.01 (120, 121). Multiple experiments or data sources for the same TF are then merged, resulting in target gene sets for 384 unique transcription factors.

**2.2. Transcription Factor Gene Expression Data**

The TF expression data used in this study was obtained from Gene Expression Barcode 3.0: http://barcode.luhs.org/index.php?page=transcriptome. HGU133plus2 (Human) tissues v3 and HGU133plus2 (Human) cells v3 are data files are used for tissue and cell line TF expression respectively.

**3. Supplemental Figures**

Figure S1:

A. Transcription factor coverage by three sources of TF-target gene data. LTP: low throughput experiments; ENCODE: TF ChIP-seq experiments in ENCODE study; Other ChIP-seq: non-ENCODE ChIP-seq experiments compiled from GEO.

B-E. Transcription factor (TF) and target gene (TG) degree distributions in the TFTG data compendium and the subset of TF-target TF core network. (B) TG degree (i.e. node in-degree) and (C) TF degree (i.e. node out-degree) distributions in the full TFTG data compendium. (D) TG degree (i.e. node in-degree) and (E) TF degree (i.e. node out-degree) distributions in the core TRTG data compendium for which the TGs are restricted to be only the 384 TFs.

Figure S2:

Venn diagram showing the overlaps among five types of gene function annotations. Mendelian diseases are not included in the Venn diagram due to limitation of the visualization technique.

Figure S3:

The significance of TF-target function association depends on the choice of gene universe. Shown here is the impact of gene universe on the association between TF SP1 and Reactome pathway *Immune System*. Three gene universes are evaluated, while the literature rich gene universe is the one used in this study.

Figure S4:

A. The in-degree and out-degree distributions of the TF-Target Function network follow a power law distribution with exponents 1.2 approximately. Left: distribution of the out-degrees of TFs (#Functions per TF). Right: distribution of the in-degrees of functions (#TFs per function).

B. The TFs that are not annotated with any target functions (blue curve) have less target genes than TFs that are annotated with one or more target functions.

Figure S5:

A. Average number of transcription factors shared per pair of function annotations. FDR cutoff 0.05 was applied to select significant target functions. The clustering is based on 1 – diagonal-normalized TF sharing, which is 1-average(cosine similarity) of regulator vectors of functional concepts.

B. Shown are heatmap of 62 TFs with significant annotation type biases at q-value cutoff 0.01. Clustering are based on cosine similarity of the counts of significant TF-Target function associations. The color in the heatmap correspond to log(Cij*C++/Ci+*C+j), where Cij is the counts for TF i and function type j, C++ is the total count, Ci+ is the total number of significant target functions for TF i, and C+j is the total number of significant TFs for a function type j. Positive values (red) indicate favored function types, and negative values (all set to -1, green color) indicate disfavored function types.

Figure S6:

Receiver Operator Characteristics (ROC) curves measuring the predictive performances of TF-Target function associations (phi) against known TF-function annotations. AUC, area under the ROC curve; FET, Fisher’s Exact Test; Centered p-value, 2 times the min of the two single tailed FET p-values.

Figure S7:

Clustering of TFs by target gene-based TF-TF similarity (measured by Pearson’s phi). In the heatmap, colors green to red correspond to negative to positive TF-TF target gene associations. Hierarchical clustering of TFs is performed using 1-phi as distance measure. Six selected sub-trees in the clustering dendrogram are shown (see **Figure S8** for a larger version of the full dendrogram).

Figure S8:

Evolutionary and functional trees of 384 TFs covered by the TFTG data compendium.

(A) Protein sequence based phylogenetic trees of TFs. Sequence similarity based clustering of transcription factors using UPGMA.

(B) Target-gene similarity (phi) based functional clustering of transcription factors, same as the clustering shown in Figure 6.

Figure S9:

Functional concepts that share TFs do not necessarily share member genes significantly.

(A) Two target functional concepts with 100% member gene overlaps always have identical regulators, but (B) two functional concepts with 100% identical regulators do not always have high member gene sharing.

Figure S10:

Significant association exists between the regulator diversity and functional diversity of 11345 genes that have both regulators and function annotations.

(A) Six types of function annotations are analyzed separately and significant positive association is observed for biological processes and signaling/metabolic pathways.

(B) Estimated odds ratio for tightly regulated genes (number of TF >= 10) versus annotated genes (number of annotation > 0).

Figure S11:

The upstream regulators and downstream (target) functions of TFs (A) NFKB1 and (B) RFX5, and non-TF gene MTHFR. The coloring schema is same as in Figure 5 and the clustering of TFs and functions are based on the target gene and member gene overlaps.

Figure S12:

Transcription factor’s regulator diversity is associated with target function diversity. Pearson correlation is 0.32, p-value 3.3e-10; and for the TF with over 100 target genes, correlation is 0.35, p-value 6.0e-06. HNF1A is highlighted in red.

**4. Supplemental Tables**

Table S1: A summary of the total number and effective number of concepts in each of the 6 function annotation databases. Phenotype: complex phenotypes studied in GWAS; Diseases: Mendelian Diseases; PharmGKB: PK & PD pathways from PharmGKB; MF: Molecular Functions from GO; BP: Biological processes from GO; Pathways: Signaling & Metabolic Pathways from Reactome.

|  | **Phenotype** | **Disease** | **PharmGKB** | **MF** | **BP** | **Reactome** |
| --- | --- | --- | --- | --- | --- | --- |
| Functional Concept Counts | 573 | 1156 | 91 | 396 | 825 | 674 |
| Within DB Redundancy-Adjusted Counts | 467.7 | 551 | 44.9 | 176.8 | 225.2 | 217.3 |
| Between DB Redundancy-Adjusted Counts | 368.4 | 465.3 | 24.8 | 115.3 | 178.7 | 163.3 |

Table S2. Average number of significant functional concept enrichments per transcription factors at FDR 0.05. Coding gene universe is used for phenotypes and literature rich gene universe for the rest.

|  | **Phenotype** | **Disease** | **PharmGKB** | **MF** | **BP** | **Reactome** |
| --- | --- | --- | --- | --- | --- | --- |
| **# Enrichment** | 0.052 | 0.471 | 0.263 | 1.383 | 7.992 | 11.216 |

Table S3: TFs and their target complex phenotypes at FDR 0.05. The Coding gene universe is used for the association analysis. Only enrichment (positive associations) and the corresponding single tailed p-values are shown. log2(OR): log2 transformed odds ratio. Evidence: lists published genetic evidence directly supports the association of the TF with the disease; Mutation: mutations of the TF are observed in the disease or closely related diseases. Association: The TF gene locus is genetically associated with the disease or related diseases. Mouse: mouse model shows phenotypes directly related to the disease. Non-genetic evidence in the literature is not considered. ^#^ MHC II: RFX5 is mutated in Bare lymphocyte syndrome II (51, 52), and RFX5 knock out mouse shows abnormality in the immune system (53, 54)

| TF | Phenotype | log_2_(OR) | P-value | Evidence |
| --- | --- | --- | --- | --- |
| **AHR** | Coffee consumption | 12.5 | 2.3E-07 | Association (55–57) |
|  | Habitual caffeine consumption | 10.1 | 5.0E-06 | Association (55–57) |
| **HNF1A** | Serum cell-free DNA (Cardiovascular Risk) | 8.9 | 1.3E-09 | Association (58),  Mutation (59) |
|  | Sickle-cell anemia (F-cell levels)  Serum bilirubin levels (bilirubin and cholelithiasis risk) | 7.6  5.7 | 5.6E-10  3.8E-06 | -  Mouse (60) |
| **NFKB1** | Psoriasis | 3.6 | 1.1E-07 | - |
|  | Ulcerative colitis | 3.3 | 5.1E-07 | Association (61) |
|  | Rheumatoid arthritis | 3.2 | 4.0E-09 | - |
| **POU2F2** | Celiac disease | 4.2 | 4.7E-06 | - |
| **RFX5** | Chronic hepatitis b | 7.1 | 8.2E-06 | - |
|  | Nasopharyngeal neoplasms | 6.5 | 9.5E-07 | - |
|  | Systemic scleroderma | 6.1 | 2.8E-10 | MHC II ^#^ |
|  | Leprosy | 4.7 | 7.5E-10 | - |
|  | Psoriasis | 3.7 | 4.9E-06 | MHC II ^#^ |
|  | Type 1 diabetes mellitus | 3.7 | 1.9E-09 | MHC II ^#^ |
|  | Multiple sclerosis | 3.3 | 2.4E-08 | MHC II ^#^ |
|  | Behcet syndrome | 3.3 | 8.8E-06 | MHC II ^#^ |
|  | Systemic lupus erythematosus | 3.2 | 1.0E-06 | MHC II ^#^ |
| **SREBF1** | Low density lipoproteins (LDL) | 3.9 | 2.2E-06 | Mouse (62) |
| **TBP** | Systemic scleroderma | 4.8 | 5.9E-06 | - |

Table S4: TF and their target pharmacogenomic pathways at FDR 0.05. The Literature Rich gene universe is used for the association detection. Log_2_(OR), log_2_ transformed odds ratio; P-value: p-value from single-tailed Fisher’s exact test for odds ratio > 1; Evidence: For the PD pathways, we listed direct evidence supporting the TF-PD pathway relationship if, 1) Member: the TF is annotated as a member gene in same or closely related PharmGKB/Reactome pathways or GO biological processes; 2) Census or Mutation: the TF is a known causal gene of the diseases based on Cancer Gene Census (92) or OMIM (49); 3) GWAS: the TF gene locus is strongly associated with related phenotypes in GWAS. 4) Mouse: mouse model shows phenotypes directly related to the disease. Other forms of evidence in the literature are not considered. ^*^ PD pathways containing 50% or more PK genes. ^&^ Neural normal: this is not disease related, but a normal pathway in neurological processes. N/A: not applicable.

| **TF** | **Pharmacogenomics pathways** | **log_2_(OR)** | **P-value** | **Diseases** | **Evidence** |
| --- | --- | --- | --- | --- | --- |
| AHR | Amodiaquine Pathway (PK) | 11.6 | 8.4E-07 | N/A | N/A |
|  | Warfarin Pathway (PK) | 9.8 | 7.9E-06 | N/A | N/A |
|  | Estrogen Metabolism Pathway | 9.6 | 4.9E-08 | N/A | N/A |
|  | Erlotinib Pathway (PK) | 9.6 | 1.0E-05 | N/A | N/A |
|  | Phenytoin Pathway (PK) | 8.3 | 5.3E-05 | N/A | N/A |
| ATF1 | Busulfan Pathway (PD) | 4.0 | 6.9E-05 | Cancer | Mutation: melanoma (151) |
| ATF2 | ACE Inhibitor Pathway (PD) | 6.3 | 3.0E-05 | Hypertension | - |
|  | Agents Acting on the Renin-Angiotensin System Pathway (PD) | 6.3 | 3.0E-05 |  |  |
| BRF1 | Imatinib Pathway (PK & PD) | 8.0 | 8.0E-05 | Cancer | - |
| BRF2 | Imatinib Pathway (PK & PD) | 7.7 | 1.2E-04 | Cancer | - |
| CREB1 | Sympathetic Nerve Pathway (Neuroeffector Junction) | 4.2 | 5.4E-06 | Neural normal^&^ | Member: Reactome REACT_18334.1, REACT_15370.2 |
| E2F1 | Antimetabolite Pathway - Folate Cycle (PD) | 4.8 | 2.5E-07 | Cancer & autoimmune diseases  Cancer & autoimmune diseases  Cancer & autoimmune diseases | Member: multiple GO terms for Cell cycle control  Member: multiple GO terms for Cell cycle control  Member: multiple GO terms for Cell cycle control |
|  | Thiopurine Pathway (PK & PD) | 3.1 | 3.2E-05 |  |  |
|  | Methotrexate Pathway (Cancer Cell) (PD) | 3.1 | 1.3E-04 |  |  |
| E2F4 | Antimetabolite Pathway - Folate Cycle (PD) | 3.5 | 2.7E-05 | Cancer & autoimmune diseases | Member: multiple GO terms for Cell cycle control |
| EGR1 | EGFR Inhibitor Pathway (PD) | 2.6 | 4.5E-05 | Cancer | - |
| ELK1 | EGFR Inhibitor Pathway (PD) | 5.6 | 5.8E-06 | Cancer | Member: PharmGKB (EGFR Inhibitor Pathway) |
| ETS1 | Vinka Alkaloid Pathway (PK) | 4.9 | 6.2E-05 | N/A | N/A |
|  | Doxorubicin Pathway (Cancer Cell) (PD) | 4.1 | 5.2E-05 | Cancer | Member: PharmGKB (EGFR Inhibitor Pathway)  Member: PharmGKB (EGFR Inhibitor Pathway) |
|  | Vemurafenib Pathway (PD) | 3.9 | 2.2E-05 | Cancer (melanoma) |  |
|  | Platelet Aggregation Inhibitor Pathway (PD) | 3.2 | 2.0E-05 | thrombosis | - |
| ETV4 | ACE Inhibitor Pathway (PD) | 5.6 | 1.1E-04 | hypertension | - |
|  | Agents Acting on the Renin-Angiotensin System Pathway (PD) | 5.6 | 1.1E-04 |  |  |
|  | Celecoxib Pathway (PD) | 4.6 | 8.2E-06 | pain | - |
| FOS | Platinum Pathway (PK & PD) | 4.4 | 3.2E-05 | Cancer  Cancer | Member: PharmGKB (EGFR Inhibitor Pathway)  Member: PharmGKB (EGFR Inhibitor Pathway) |
|  | Doxorubicin Pathway (Cancer Cell) (PD) | 4.1 | 1.3E-05 |  |  |
| FOXA1 | Benzodiazepine Pathway (PK) | 4.6 | 1.1E-04 | N/A | N/A |
| FOXA2 | Carbamazepine Pathway (PK) | 4.4 | 2.1E-05 | N/A | N/A |
|  | Phenytoin Pathway (PK) | 4.0 | 7.0E-05 | N/A | N/A |
| HIF1A | Glucocorticoid Pathway (Peripheral Tissue) (PD) | 6.0 | 6.3E-05 | Inflammation | Member: GO cytokine production |
| HNF1A | Artemisinin and Derivatives Pathway (PK) | 8.4 | 5.4E-07 | N/A | N/A |
|  | Tramadol (PK) | 8.1 | 9.4E-07 | N/A | N/A |
|  | Losartan Pathway (PK) | 8.0 | 9.2E-05 | N/A | N/A |
|  | Sorafenib(PK) | 7.6 | 1.4E-04 | N/A | N/A |
|  | Mycophenolic acid Pathway (PK) | 7.4 | 1.2E-09 | N/A | N/A |
|  | Irinotecan Pathway (PD*) | 7.3 | 7.4E-08 | Cancer | Mutation: renal cell carcinoma (93) |
|  | Benzodiazepine Pathway (PK) | 7.3 | 4.4E-06 | N/A | N/A |
|  | Valproic Acid Pathway (PK) | 7.0 | 4.0E-09 | N/A | N/A |
|  | Irinotecan Pathway (PK) | 7.0 | 7.6E-06 | N/A | N/A |
|  | Tamoxifen Pathway (PK) | 6.9 | 2.3E-07 | N/A | N/A |
|  | Estrogen Metabolism Pathway | 6.7 | 1.2E-05 | N/A | N/A |
|  | Phenytoin Pathway (PK) | 6.3 | 3.0E-05 | N/A | N/A |
|  | Statin Pathway (PD) | 5.9 | 5.9E-05 | High cholesterol | Association (71) |
|  | Anti-diabetic Drug Potassium Channel Inhibitors Pathway (PD) | 5.7 | 8.4E-05 | Diabetes | Mutation: diabetes, type 1 (94) and type 2 (95) |
| HNF1B | Methotrexate Pathway (PK) | 9.3 | 1.5E-05 | N/A | N/A |
| HNF4G | Statin Pathway (PD) | 4.7 | 6.5E-05 | High cholesterol | Weak Association (96) |
| HOXA5 | Doxorubicin Pathway (Cancer Cell) (PD) | 8.2 | 6.0E-05 | Cancer | Mouse: abnormal cell migration (97) |
| HSF1 | Glucocorticoid Pathway (Peripheral Tissue) (PD) | 7.1 | 6.4E-06 | Inflammation | Mouse (98) |
| KLF11 | Anti-diabetic Drug Potassium Channel Inhibitors Pathway (PD) | 9.2 | 2.1E-05 | Diabetes | Mutation (99) |
| MYC | Antimetabolite Pathway - Folate Cycle (PD) | 3.9 | 4.5E-06 | Cancer & autoimmune diseases | Member: GO cell cycle control;  Census (100, 101); |
|  | Thiopurine Pathway (PK & PD) | 2.4 | 8.6E-05 | Cancer & autoimmune diseases | Member: GO cell cycle control;  Census (100, 101); |
| NFKB1 | Doxorubicin Pathway (Cancer Cell) (PD) | 4.4 | 3.8E-06 | Cancer  Cancer | - (Strong literature evidence but no mutation)  - |
|  | EGFR Inhibitor Pathway (PD) | 3.1 | 8.9E-07 |  |  |
| NR1I2 | Clopidogrel Pathway (PK) | 6.1 | 4.7E-05 | N/A | N/A |
| NR1I3 | Fluvastatin Pathway (PK) | 11.3 | 1.5E-06 | N/A | N/A |
|  | Atorvastatin/Lovastatin/Simvastatin Pathway (PK) | 11.2 | 1.7E-06 | N/A | N/A |
|  | Statin Pathway – Generalized (PK) | 10.7 | 3.6E-06 | N/A | N/A |
|  | Phenytoin Pathway (PK) | 10.7 | 3.6E-06 | N/A | N/A |
| NR2C2 | Imatinib Pathway (PK & PD) | 7.7 | 1.2E-04 | Cancer | - |
| PDX1 | Anti-diabetic Drug Potassium Channel Inhibitors Pathway (PD) | 9.2 | 2.1E-05 | Diabetes | Mutation (102, 103) |
| PHOX2A | Sympathetic Nerve Pathway (Neuroeffector Junction) | 9.9 | 9.9E-06 | Neural normal^&^ | Mouse (104) |
| PPARA | Statin Pathway (PD) | 6.3 | 3.8E-08 | High cholesterol | Mutation (105) |
|  | Celecoxib Pathway (PD) | 4.6 | 7.8E-05 | Pain | - |
| PPARD | Statin Pathway (PD) | 6.4 | 2.1E-05 | High cholesterol | Mouse (106) |
| RARA | Aromatase Inhibitor Pathway (Breast Cell) (PD) | 8.1 | 1.6E-06 | Cancer  Cancer | Census  Census |
|  | EGFR Inhibitor Pathway (PD) | 4.0 | 1.1E-05 |  |  |
| RARB | Vemurafenib Pathway (PD) | 5.6 | 1.0E-04 | Cancer  Cancer | -  - |
|  | EGFR Inhibitor Pathway (PD) | 4.5 | 1.0E-04 |  |  |
| RDBP | Imatinib Pathway (PK & PD) | 8.0 | 8.0E-05 | Cancer | - (Involved in cancer, not through genetic mutation) |
| REST | Sympathetic Nerve Pathway (Pre- and Post- Ganglionic Junction) | 4.9 | 6.9E-05 | Neural normal^&^ | Mouse: thick retinal ganglion layer (107) |
| RXRA | Statin Pathway (PD) | 5.4 | 6.1E-08 | High cholesterol | - |
| SP1 | Vinka Alkaloid Pathway (PK) | 4.6 | 5.2E-06 | N/A | N/A |
|  | Erlotinib Pathway (PK) | 4.3 | 5.7E-05 | N/A | N/A |
|  | Etoposide Pathway (PK & PD) | 3.9 | 7.3E-06 | Cancer | - |
|  | Statin Pathway (PD) | 3.8 | 3.4E-08 | High cholesterol | - |
|  | EGFR Inhibitor Pathway (PD) | 2.1 | 7.3E-05 | Cancer | - |
| SREBF1 | Bisphosphonate Pathway (PD) | 5.7 | 3.0E-07 | Osteoporosis | - |
|  | Statin Pathway (PD) | 5.3 | 7.0E-08 | High cholesterol | Mouse Knockout (108) |
| SREBF2 | Bisphosphonate Pathway (PD) | 6.8 | 9.3E-09 | Osteoporosis | - |
|  | Statin Pathway (PD) | 6.4 | 1.1E-09 | High cholesterol | Mouse Knockout (108) |
| STAT1 | EGFR Inhibitor Pathway (PD) | 3.1 | 1.9E-05 | Cancer | - |
| STAT5A | Aromatase Inhibitor Pathway (Breast Cell) (PD) | 8.9 | 2.8E-05 | Breast & ovarian cancer | - |
| STAT5B | Aromatase Inhibitor Pathway (Breast Cell) (PD) | 8.9 | 2.6E-05 | Breast & ovarian cancer | Cancer Gene Census |
| TFAP2A | Sympathetic Nerve Pathway (Neuroeffector Junction) | 3.6 | 2.4E-06 | Neural normal^&^ | Mutation: Branchio-oculo-facial syndrome (109–111)  - |
|  | Celecoxib Pathway (PD) | 3.2 | 4.3E-07 | Pain |  |
|  | EGFR Inhibitor Pathway (PD) | 2.6 | 8.8E-05 | Cancer |  |
| TP53 | Doxorubicin Pathway (Cancer Cell) (PD) | 5.2 | 2.0E-09 | Cancer | Census (112, 113) |
|  | Vinka Alkaloid Pathway (PK) | 5.0 | 4.6E-05 | - | - |
|  | Etoposide Pathway (PK & PD) | 4.6 | 1.3E-05 | Cancer | Census (112, 113) |
|  | Busulfan Pathway (PD) | 4.2 | 3.9E-09 | Cancer | Census (112, 113) |
|  | Vemurafenib Pathway (PD) | 4.0 | 1.5E-05 | Cancer | Census (112, 113) |
|  | Methotrexate Pathway (Cancer Cell) (PD) | 4.0 | 2.6E-06 | Cancer & autoimmune diseases | Census (112, 113); Normal immune response (114) |
|  | Doxorubicin Pathway (PK) | 3.9 | 1.0E-04 | - | - |
|  | EGFR Inhibitor Pathway (PD) | 3.4 | 2.4E-08 | Cancer | Census (112, 113) |
| USF1 | Theophylline Pathway (PK) | 6.0 | 1.2E-04 | - | - |
| WT1 | Doxorubicin Pathway (Cancer Cell) (PD) | 6.6 | 4.6E-07 | Cancer | Census (115) |
|  | Doxorubicin Pathway (PK) | 5.8 | 6.7E-05 | - | - |
|  | Vemurafenib Pathway (PD) | 5.6 | 1.0E-04 | Cancer | Census (115) |
| YBX1 | Erlotinib Pathway (PK) | 9.9 | 6.7E-06 | - | - |

**Table S5.** The tables list the functional concepts with the highest and lowest unique scores from 6 sources of gene function annotations. The uniqueness score are computed with all data sources merged.

| GWAS Phenotypes | Uniqueness | Mendelian Diseases | Uniqueness |
| --- | --- | --- | --- |
| oligospermia | 1 | anauxetic dysplasia | 1 |
| cd8-positive t-lymphocytes | 1 | polycystic ovary syndrome | 1 |
| t-lymphocytes | 1 | intracranial arterial disease | 1 |
| serotonin | 1 | Moyamoya disease | 1 |
| transforming growth factor beta1 | 1 | Mobius syndrome | 1 |
| lymphoma, follicular | 0.24 | papilloma | 0.1 |
| hepatitis b, chronic | 0.24 | choroid plexus papilloma | 0.1 |
| myeloproliferative disorders | 0.23 | adrenocortical carcinoma | 0.1 |
| alopecia | 0.22 | adrenal gland cancer | 0.1 |
| papillomaviridae | 0.18 | adrenal cortex cancer | 0.1 |

| PharmGKB PK/PD pathways | Uniqueness | GO MF | Uniqueness |
| --- | --- | --- | --- |
| PA165947317_Leukotriene_modifiers_pathway_PD | 0.75 | DRUG_BINDING | 0.76 |
| PA2026_Glucocorticoid_Pathway_(HPA_Axis)_PD | 0.73 | PROTEIN_TRANSPORTER_ACTIVITY | 0.74 |
| PA165111376_Benzodiazepine_Pathway_PD | 0.66 | OXIDOREDUCTASE_ACTIVITY_ACTING_ON_SULFUR_GROUP_OF_DONORS | 0.72 |
| PA2027_Glucocorticoid_Pathway_(Peripheral_Tissue)_PD | 0.65 | OXIDOREDUCTASE_ACTIVITY_ACTING_ON_THE_CH_NH_GROUP_OF_DONORS | 0.7 |
| PA2036_Gemcitabine_Pathway_PD | 0.59 | NUCLEOBASENUCLEOSIDENUCLEOTIDE_AND_NUCLEIC_ACID_TRANSMEMBRANE_TRANSPORTER_ACTIVITY | 0.68 |
| PA145011111_Fluvastatin_Pathway_PK | 0.11 | METAL_ION_TRANSMEMBRANE_TRANSPORTER_ACTIVITY | 0.08 |
| PA152325160_Gefitinib_Pathway_PK | 0.1 | ION_CHANNEL_ACTIVITY | 0.08 |
| PA2034_Cyclophosphamide_Pathway_PK | 0.1 | TRANSMEMBRANE_TRANSPORTER_ACTIVITY | 0.08 |
| PA145011108_Statin_Pathway_-_Generalized_PK | 0.1 | ION_TRANSMEMBRANE_TRANSPORTER_ACTIVITY | 0.07 |
| PA145011109_Atorvastatin/Lovastatin/Simvastatin_Pathway_PK | 0.09 | SUBSTRATE_SPECIFIC_TRANSMEMBRANE_TRANSPORTER_ACTIVITY | 0.07 |

| GO BP | Uniqueness | Signaling/Metabolic Pathways | Uniqueness |
| --- | --- | --- | --- |
| ESTABLISHMENT_AND_OR_MAINTENANCE_OF_CELL_POLARITY | 0.63 | TRYPTOPHAN_CATABOLISM | 0.86 |
| KERATINOCYTE_DIFFERENTIATION | 0.58 | DIGESTION_OF_DIETARY_CARBOHYDRATE | 0.76 |
| RESPIRATORY_GASEOUS_EXCHANGE | 0.58 | VITAMIN_B5_PANTOTHENATE_METABOLISM | 0.72 |
| PEPTIDE_METABOLIC_PROCESS | 0.58 | METABOLISM_OF_POLYAMINES | 0.72 |
| MEMBRANE_FUSION | 0.58 | CELL_EXTRACELLULAR_MATRIX_INTERACTIONS | 0.7 |
| REGULATION_OF_TRANSCRIPTION | 0.06 | CDT1_ASSOCIATION_WITH_THE_CDC6_ORC_ORIGIN_COMPLEX | 0.04 |
| REGULATION_OF_NUCLEOBASENUCLEOSIDENUCLEOTIDE_AND_NUCLEIC_ACID_METABOLIC_PROCESS | 0.06 | P53_INDEPENDENT_G1_S_DNA_DAMAGE_CHECKPOINT | 0.04 |
| REGULATION_OF_GENE_EXPRESSION | 0.06 | SCF_BETA_TRCP_MEDIATED_DEGRADATION_OF_EMI1 | 0.04 |
| REGULATION_OF_METABOLIC_PROCESS | 0.06 | AUTODEGRADATION_OF_THE_E3_UBIQUITIN_LIGASE_COP1 | 0.04 |
| REGULATION_OF_CELLULAR_METABOLIC_PROCESS | 0.06 | CDK_MEDIATED_PHOSPHORYLATION_AND_REMOVAL_OF_CDC6 | 0.04 |

**Table S6:** A list of negatively associated functional concepts regulated by shared transcription factors. Negative association of two concepts is defined as a negative Phi coefficient defined based on the member genes of two functional concepts A and B.

**File:** TableS6.xlsx

**Table S7**: The complete TF annotation results. -Log_10_ (p-value) are provided in parentheses following the target functions.

**File:** TableS7.xlsx

**Table S8**: The complete list of TF pairs with significant target function overlaps but lower than expected target gene overlaps. Negative association (i.e. lower than expected target gene overlaps) of two TFs is defined as a negative Phi coefficient of the target gene overlaps of two TFs TF1 and TF2.

**File:** TableS8.xlsx

Table S9. Discordance transcription factors’ target function similarity and target gene similarity. Significant target-function sharing: target function overlap significantly higher than expected by change (FDR <= 0.05). High sharing of target gene: odds ratio of target gene sharing between a pair of TF is >= 1. Significant sharing of known functions: known function overlap significantly higher than expected by change (FDR <= 0.05). OR: odds ratio.

| TF pair classification | | | Counts | Significant sharing of known functions | P-value |
| --- | --- | --- | --- | --- | --- |
| Description | **Target-function sharing** | **Target-gene sharing** |  |  |  |
| Unexpected target function similarity | Significant | Low | 329 | 124 (37.7%) | 0.011  OR = 1.31 |
| Other pairs with low target gene sharing | Not | Low | 42373 | 13375 (31.6%) |  |
| Expected target function similarity | Significant | High | 4583 | 1942 (42.4%) | 2.6E-12 OR = 1.25 |
| Other pairs with high target gene sharing | Not | High | 26251 | 9706 (37.0%) |  |
